# Proportions of *Staphylococcus aureus* and Methicillin-Resistant *Staphylococcus aureus* in Patients with Surgical Site Infections in Mainland China: A Systematic Review and Meta-Analysis

**DOI:** 10.1371/journal.pone.0116079

**Published:** 2015-01-20

**Authors:** Zhirong Yang, Jing Wang, Weiwei Wang, Yuelun Zhang, Lizhong Han, Yuan Zhang, Xiaolu Nie, Siyan Zhan

**Affiliations:** 1 Department of Epidemiology and Biostatistics, School of Public Health, Peking University, Beijing, China; 2 Shantou-Oxford Clinical Research Unit, Shantou University Medical College, Shantou, Guangdong, China; 3 Department of Clinical Microbiology, Ruijin Hospital, Shanghai Jiao Tong University School of Medicine, Shanghai, China; 4 Department of Clinical Epidemiology and Biostatistics, McMaster University, Hamilton, Ontario, Canada; University of Massachusetts, UNITED STATES

## Abstract

**Background:**

Sufficient details have not been specified for the epidemiological characteristics of *Staphylococcus aureus* (*S. aureus*) and methicillin-resistant *Staphylococcus aureus* (MRSA) among surgical site infections (SSIs) in mainland China. This systematic review aimed to estimate proportions of *S. aureus* and MRSA in SSIs through available published studies.

**Methods:**

PubMed, Embase and four Chinese electronic databases were searched to identify relevant primary studies published between 2007 and 2012. Meta-analysis was conducted on the basis of logit-transformed metric for proportions of *S. aureus* and MRSA, followed by pre-defined subgroup meta-analysis. Random-effects meta-regression was also conducted to explore the impact of possible factors on *S. aureus* proportions.

**Results:**

106 studies were included, of which 38 studies involved MRSA. *S. aureus* accounted for 19.1% (95%CI 17.2-21.0%; I^2^ = 84.1%) of all isolates in SSIs, which was roughly parallel to 18.5% in the United States (US) (P-value = 0.57) but significantly exceeded those calculated through the surveillance system in China (P-value<0.001). In subgroup analysis, *S. aureus* in patients with thoracic surgery (41.1%, 95%CI 26.3-57.7%; I^2^ = 74.4%) was more common than in those with gynecologic surgery (20.1%, 95%CI 15.6-25.6%; I^2^ = 33.0%) or abdominal surgery (13.8%, 95%CI 10.3-18.4%; I^2^ = 70.0%). Similar results were found in meta-regression. MRSA accounted for 41.3% (95%CI 36.5-46.3%; I^2^ = 64.6%) of *S. aureus*, significantly lower than that in the US (P-value = 0.001). MRSA was sensitive to vancomycin (522/522) and linezolid (93/94), while 79.9% (95%CI 67.4-88.4%; I^2^ = 0%) and 92.0% (95%CI 80.2-97.0%; I^2^ = 0%) of MRSA was resistant to clindamycin and erythromycin respectively.

**Conclusion:**

The overall proportion of *S. aureus* among SSIs in China was similar to that in the US but seemed higher than those reported through the Chinese national surveillance system. Proportions of *S. aureus* SSIs may vary with different surgery types. Commonly seen in SSIs, MRSA tended to be highly sensitive to vancomycin and linezolid but mostly resistant to clindamycin and erythromycin.

## Introduction

It has been widely accepted that surgical site infections (SSIs) are an important component of all the nosocomial infection. Three types of SSIs are defined by the Centers for Disease Control and Prevention (CDC), including superficial, deep incisional SSIs and organ-space SSIs, depending on the sites and the extent of infection [1], among which superficial incisional SSIs are more common than the other two types [2]. In the United States (US) 2%–5% of patients undergoing surgeries develop SSIs of varying severity [3]. In studies from Europe, SSIs have a similar incidence, hovering at 3%–5% among patients undergoing surgery [4, 5]. SSIs are associated with increased morbidity and mortality rate [6]. In addition, patients with SSIs have a heavy economic burden in terms of extended length of stay and increased costs of treatment [7].

Various pathogens can contribute to SSIs, but significant concern has been raised for *Staphylococcus aureus* (*S. aureus*) and methicillin-resistant *Staphylococcus aureus* (MRSA). As the primary pathogen, *S. aureus* constitute approximately 20% of SSIs cases among hospitals according to the CDC [8]. From 1992 to 2002 the proportion of SSIs caused by *S. aureus* increased from 16.6% to 30.9%, during which time MRSA isolates increased from 9.2% to 49.3% [9]. The 90 days post-operative mortality was 6.7% and 20.7% for SSIs patients with methicillin-susceptible *S. aureus* (MSSA) and MRSA, respectively [10]. Compared with MSSA, the additional hospital charge associated with MRSA was at least $40,000 [10].

In China, the proportions of *S. aureus* in SSIs have been available from the Nosocomial Infection Surveillance System since 1990s; however, the system which covers a wide range of nosocomial infections is not specific to SSIs and the statistics reported from the system to date remain far from sufficient to describe the epidemiology of *S. aureus* or MRSA in SSIs across the country. Up to now, only three studies [11–13] have been published on the basis of this system, reporting the proportion of *S. aureus* in SSIs. According to the data from 79 hospitals in the system *S. aureus* accounted for 12.7% (377/2,971) between 1999 and 2001 [11] and 13.5% (515/3812) between 1999 and 2005 [12] among pathogens in SSIs. The proportions of *S. aureus* in patients with superficial incisional, deep incisional and organ-space infections were 14.1%, 12.8% and 7.4% respectively between 1999 and 2007 based on the data from 110 hospitals [13]. Furthermore, several limitations of the publications from this system existed. Firstly, the selected hospitals in the studies seemed unable to represent all the nationwide hospitals. The number of hospitals within the system had amounted to 134 in 2001 [14], but none of the studies involved all or a random sample of the hospitals to estimate the proportion of *S. aureus* in SSIs patients, which may introduce selection bias. Secondly, the exact data about the proportions of MRSA and the proportions of drug resistance of MRSA in SSIs, which should be the main concern from the perspective of clinical practice, are not accessible in the studies. In addition, this system only provided the overall proportion of *S. aureus* rather than proportions by year, region, hospital level, and surgery type, which are likely to have more significant impact on decision-making for clinical practice and public health.

Understanding the nationwide epidemiological situation of *S. aureus* and MRSA in SSIs is vital for policy makers and clinicians to develop appropriate preventive countermeasures. As the national data in China remain inadequate, this systematic review aimed to estimate the proportions of *S. aureus* and MRSA in SSIs, by summarizing and assessing the available observational studies in China published from 2007 to 2012, to provide further evidence.

## Methods

### Information sources and search strategy

We performed systematic search in six electronic databases, including PubMed, Embase (OVID), Chinese BioMedical Database (CBM), China National Knowledge Infrastructure (CNKI), VIP Chinese Science and Technique Journals Database, and Wanfang Database, to identify the relevant studies. Since the focus in the review was on the epidemiological characteristics of *S. aureus* and MRSA in SSIs during recent years, search was limited to the publication date from January 2007 to November 2012. A combination of Mesh words and free text words applied to PubMed, Embase and CBM, and free text words were used to search CNKI, VIP and Wanfang database. The following search terms were mainly used: “surgery”, “wound infection*”, “postoperative wound infection*”, “surgical site infection*”, “*S. aureus*.”, “*Staphylococcus aureus*”, “methicillin”, “MSSA” and “MRSA”. Details of the search strategies for each database were summarized in S1 Table.

### Eligibility criteria

Criteria of inclusion:

- Patients: those with SSIs regardless of other characteristics;
- Outcomes: *S. aureus* and MRSA isolates identified from SSIs;
- Study types: observational studies including cross-sectional, monitoring, prospective, ambispective and retrospective study.

Criteria of exclusion:

- Duplicate studies;
- Involvement of studied population from outside mainland China;
- Therapeutic study including randomized controlled trial and observational research for comparative effectiveness;
- Studies with data from the China Nosocomial Infection Surveillance System.

### Study selection

According to the criteria of inclusion and exclusion, two reviewers independently screened each record by the title, keywords and abstract. The eligibility was determined further through the full texts if selection cannot be made only based on the screening. Any disagreement was resolved by the third reviewer.

### Data extraction

An original extraction form was designed and then modified following a pilot test. The revised extraction form encompassed three parts: general information, clinical characteristics and numbers for calculating proportions of *S. aureus* and MRSA isolates. Two reviewers extracted information from each study independently. Any disagreement was also resolved by the third reviewer.

### Assessment of risk of bias

As there were no acknowledged or standardized quality assessment tools for the included study designs, we used a checklist with 8 items adapted from a scale for case series [15], which was originally developed by the National Institute for Health and Care Excellence (NICE), a special health authority in the UK which is committed to providing national guidance and advice to improve health and social care. Low, high or unclear risk of bias for each item was determined according to the pre-specified criteria (S2 Table) and the graph of summary of risk of bias was developed with Revman 5.1. One point was scored if an item was judged low risk of bias. We defined study of higher quality with a total of at least 4 points.

### Dealing with missing data

When information of the variables for analysis was missing from publications, the correspondent authors were contacted by email every one week. If the authors did not reply to the emails after our second contact attempt, the publications were excluded when the related variables were analyzed.

### Statistical analysis

We conducted all the data analyses using R (Version 3.1.2, The R Foundation for Statistical Computing).

### Calculation formula for the proportions of *S. aureus* and MRSA

Proportions of *S. aureus* and MRSA isolates were calculated by the following formula for each related study:

$$\text{Proportion of S. aureus}=\frac{\text{Number of S. aureus isolates detected}}{\text{Number of all the detected isolates}}\times 100\%$$

$$\text{Proportion of MRSA}=\frac{\text{Number of MRSA isolates detected}}{\text{Number of S.aureus isolates detected}}\times 100\%$$

$$\text{Proportion of antibiotics-resistant MRSA}=\frac{\text{Number of detected MRSA isolates resistant to a given antibiotic}}{\text{Number of MRSA isolates detected}}\times 100\%$$

Incremental 0.5 was added to both the numerator and denominator in studies with zero or all events. 95% CI for the proportion in each study was calculated based on the logit-transformed metric.

### Pooled overall proportions

Meta-analysis was conducted for the pooled estimates, followed by comparison between our overall estimate of *S. aureus* and MRSA and the corresponding proportions in the US and in the China Nosocomial Infection Surveillance System. Statistical difference between the proportions in such comparisons was tested by Q statistic for heterogeneity [16]. P-value of less than 0.05 indicated statistical significance. Considering probable heterogeneity across all the observational studies, random-effects model with Der-Simonian Laird method was used *a priori* throughout the data analyses.

### Heterogeneity and subgroup analysis

Q test and I^2^ statistic were used to examine and quantify the heterogeneity of the logit-transformed proportion across the studies. P-value of less than 0.05 or I^2^ statistic of more than 50% were regarded as substantial heterogeneity [17]. Subgroup analysis was conducted to explore the possible sources of heterogeneity based on the pre-defined variables including study quality, sample size, region, level of hospital, provincial economic condition, types of surgeries. A map for the distribution of *S. aureus* was drawn through MapInfo Professional 11.0 according to the subgroup analysis by provinces. We determined small sample size if at most 20 bacteria isolates or *S.aureus* isolates were included in analysis for primary studies respectively reporting the proportion of *S. aureus* or MRSA. Based on whether the annual Gross Domestic Product (GDP) per capita of each province in 2011 was higher or lower than the national average (35,181RMB) in China, provinces were categorized into higher or lower provincial economic condition [18].

Informal comparisons were made between subgroups for the proportions of *S. aureus* and MRSA by directly comparing the magnitudes of proportions between different subgroups instead of significance tests which tend to be misleading for the comparison in subgroup analysis. Statistical significance was defined as non-overlap of the confidence intervals of the proportions between the subgroups [19].

### Meta-regression for the proportion of *S. aureus* isolates

Meta-regression was used to explore the impact of pre-defined factors on the proportion of *S. aureus* isolates. We defined logit(*P*) as the dependent variable where *P* referred to proportion of *S. aureus* isolates. All the independent factors were initially selected based on the expertise in clinical microbiology and the availability of related information in the included articles, including study quality, sample size, region, level of hospital, provincial economic condition and type of surgery, all of which were defined as dummy variables. The factors without colinearity indicated by no correlation to each other (P-value≥0.10) were finally included into the random-effects meta-regression model with restricted maximum likelihood (REML) method. The statistical significance of any single coefficient was tested by Z-test and 0.05 was used as the threshold of P-value for statistically significant difference.

### Publication bias

Egger’s test served to assess the probability of publication bias for the overall *S. aureus* and MRSA proportion [20]. The test was based on the logit-transformed proportion and corresponding standard error. A P-value of less than 0.10 was regarded as statistical significance, indicating probable publication bias.

## Results

### General information about included studies

**Figure 1:**
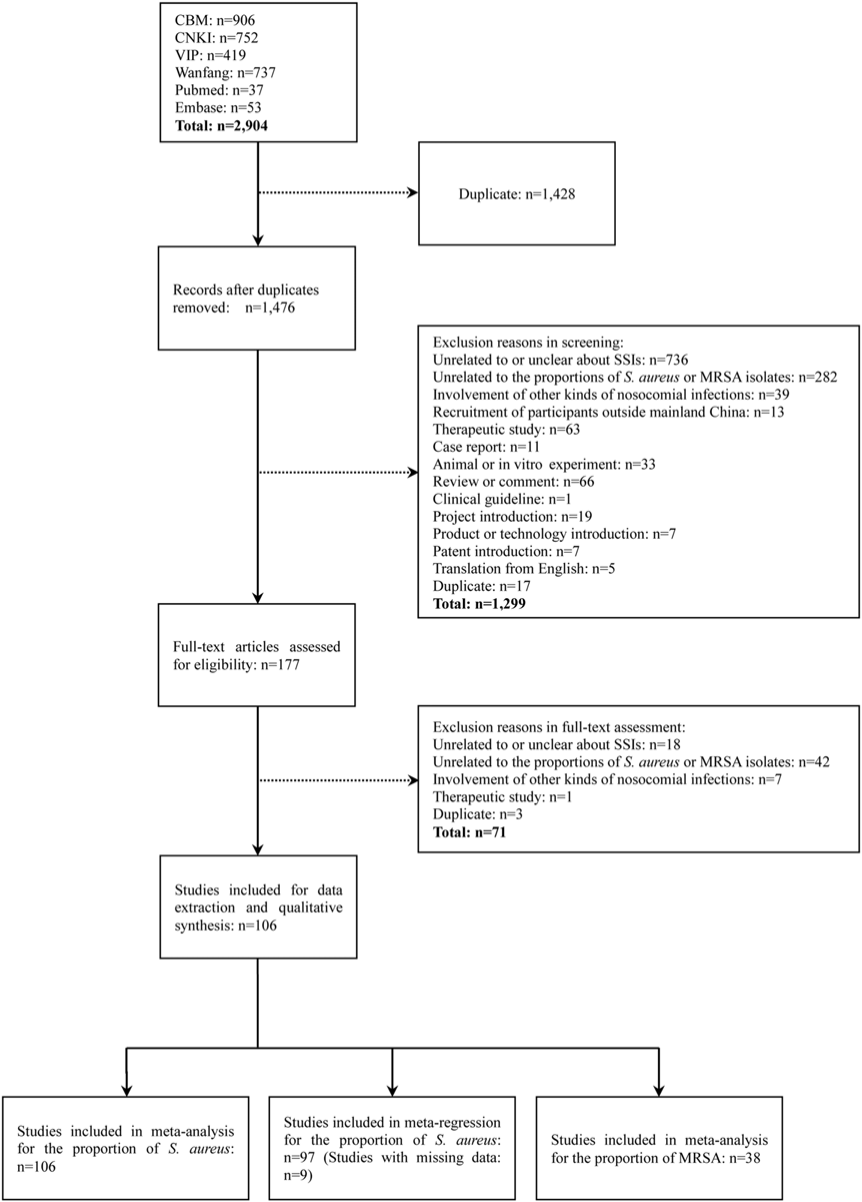
Flow diagram of study inclusion.

We retrieved 2904 references from six databases, of which 106 studies were eligible for inclusion (Fig. 1). All the studies, 105 published in Chinese and one in English, were hospital-based. Table 1, Table 2 and S3 Table show detailed characteristics of included studies.

**Table 1:** General information of all the included studies.

**Study ID**	**Province**	**Start**	**Finishing**	**Number of centers**	**Study type**	**Number of SSIs patients**	Surgery type*	**Relevance to MRSA**	**Region**	**Hospital level**	**Economy**	**Score for risk of bias**
Ao 2007 [31]	Jiangxi	March 2005	March 2007	1	Retrospective	89	Orthopedic	No	Urban	Tertiary	Lower	3
Chang 2010 [32]	Shanxi	January 2007	December 2009	1	Ambispective	95	Multiple	No	Urban	Tertiary	Lower	4
Chen 2009a [33]	Fujian	January2006	December 2008	1	Retrospective	Unclear	Orthopedic	Yes	Urban	Tertiary	Higher	2
Chen 2009b [34]	Guangdong	January 2002	December 2006	1	Prospective	Unclear	Neurosurgery	No	Urban	Tertiary	Higher	2
Chen 2010 [35]	Beijing	January 2006	December 2008	1	Retrospective	214	Multiple	Yes	Urban	Tertiary	Higher	3
Chen 2012 [36]	Fujian	January 2007	December 2010	1	Ambispective	105	Multiple	No	Rural	Non-tertiary	Higher	3
Cui 2008 [37]	Henan	January 2007	December 2007	1	Retrospective	1001	Multiple	No	Urban	Non-tertiary	Lower	2
Dai 2012 [38]	Zhejiang	February 2009	May 2011	1	Retrospective	50	Abdominal	No	Urban	Tertiary	Higher	3
Deng 2010 [39]	Sichuan	Unclear	Unclear	10	Cross-sectional	26	Multiple	Yes	Urban	Non-tertiary	Lower	3
Ding 2010 [40]	Liaoning	January 2000	December 2009	1	Retrospective	145	Multiple	No	Urban	Non-tertiary	Higher	3
Dong 2007 [41]	Zhejiang	January 2006	December 2006	1	Monitoring	Unclear	Multiple	No	Urban	Tertiary	Higher	3
Duan 2008 [42]	Hubei	January 2002	December 2007	1	Retrospective	18	Multiple	Yes	Rural	Non-tertiary	Lower	4
Fan 2008 [43]	Hubei	January 2004	December 2007	1	Retrospective	221	Abdominal	Yes	Urban	Tertiary	Lower	4
Fan 2010 [44]	Zhejiang	January 2003	December 2008	1	Retrospective	36	Orthopedic	No	Urban	Non-tertiary	Higher	2
Gu 2009 [45]	Xinjiang	January 2002	December 2006	1	Retrospective	76	Gynecologic	No	Urban	Non-tertiary	Lower	0
Hao 2012 [46]	Hebei	October 2010	December 2011	1	Unclear	61	Orthopedic	Yes	Urban	Tertiary	Lower	4
He 2012 [47]	Jiangxi	January 2009	December 2011	1	Monitoring	41	Multiple	No	Urban	Tertiary	Lower	5
Huang 2012 [48]	Liaoning	June 2008	October 2010	1	Retrospective	19	Abdominal	No	Urban	Non-tertiary	Higher	4
Jiang 2009 [49]	Guangdong	January 2003	December 2007	1	Retrospective	332	Orthopedic	No	Urban	Tertiary	Higher	2
Jiang 2012 [50]	Beijing	January 2009	December 2010	1	Monitoring	62	Gynecologic	No	Rural	Non-tertiary	Higher	6
Li 2008 [51]	Henan	December 2004	December2007	1	Retrospective	36	Gynecologic	No	Rural	Non-tertiary	Lower	2
Li 2009a [52]	Sichuan	April 2008	August 2008	1	Unclear	26	Multiple	No	Urban	Tertiary	Lower	3
Li 2009b [53]	Liaoning	January 2006	December 2008	1	Retrospective	15	Gynecologic	No	Urban	Non-tertiary	Higher	2
Li 2009c [54]	Henan	January 2007	December 2008	1	Retrospective	133	Orthopedic	No	Urban	Tertiary	Lower	4
Li 2010a [55]	Hubei	January 2005	December 2008	1	Retrospective	241	Multiple	Yes	Urban	Non-tertiary	Higher	3
Li 2010b [56]	Hunan	January 2007	December 2009	Unclear	Retrospective	106	Multiple	No	Rural	Non-tertiary	Lower	3
Li 2010c [57]	Hebei	September 2007	September 2009	1	Retrospective	139	Orthopedic	Yes	Urban	Tertiary	Lower	1
Li 2011a [58]	Guangxi	January2007	June 2010	1	Unclear	118	Multiple	Yes	Rural	Non-tertiary	Lower	4
Li 2011b [59]	Jiangsu	Unclear	Unclear	Unclear	Unclear	20	Gynecologic	No	Urban	Non-tertiary	Higher	2
Li 2012 [60]	Xinjiang	November 2009	November 2011	1	Retrospective	32	Gynecologic	No	Urban	Tertiary	Lower	4
Lin 2007 [61]	Zhejiang	January 2004	June 2004	1	Unclear	411	Multiple	No	Rural	Non-tertiary	Higher	3
Lin 2008 [62]	Guangdong	January 2004	December 2007	1	Ambispective	18	Orthopedic	No	Urban	Non-tertiary	Higher	4
Lin 2009 [63]	Fujian	January 2007	December 2008	1	Retrospective	31	Abdominal	No	Urban	Tertiary	Higher	3
Ling 2011 [64]	Zhejiang	January 2007	December 2009	1	Monitoring	224	Orthopedic	Yes	Urban	Non-tertiary	Higher	4
Liu 2008 [65]	Guangdong	January 2005	May 2007	1	Ambispective	113	Abdominal	No	Urban	Non-tertiary	Higher	4
Liu 2010 [66]	Henan	January 2006	June 2009	1	Ambispective	274	Multiple	No	Urban	Tertiary	Lower	4
Liu 2011 [67]	Tianjin	January 2005	December 2008	1	Ambispective	119	Orthopedic	No	Urban	Tertiary	Higher	3
Liu 2012a [68]	Jiangxi	January 2010	February 2012	1	Unclear	196	Thoracic	Yes	Urban	Tertiary	Lower	2
Liu 2012b [69]	Henan	March 2010	March 2011	1	Retrospective	128	Abdominal	No	Rural	Non-tertiary	Lower	3
Liu 2012c [70]	Hubei	January 2008	December 2010	1	Retrospective	213	Multiple	Yes	Rural	Non-tertiary	Lower	3
Lv 2007 [71]	Zhejiang	January 2003	May 2006	1	Retrospective	139	Multiple	No	Urban	Non-tertiary	Higher	4
Lv 2012 [72]	Zhejiang	January 2006	June 2011	1	Retrospective	26	Abdominal	No	Urban	Tertiary	Higher	3
Mao 2011 [73]	Zhejiang	June 2008	September 2010	1	Retrospective	285	Orthopedic	Yes	Urban	Tertiary	Higher	3
Pang 2007 [74]	Sichuan	January 2001	December 2004	1	Retrospective	64	Multiple	No	Rural	Non-tertiary	Lower	4
Peng 2008 [75]	Hubei	January 2004	December 2007	1	Unclear	78	Multiple	Yes	Rural	Non-tertiary	Lower	1
Peng 2012a [76]	Hubei	January 2009	December 2010	1	Retrospective	254	Multiple	Yes	Urban	Tertiary	Lower	3
Peng 2012b [77]	Hubei	January 2008	May 2011	1	Retrospective	169	Multiple	Yes	Urban	Tertiary	Lower	3
Qian 2011 [78]	Zhejiang	January 2000	December 2009	1	Retrospective	135	Gynecologic	Yes	Urban	Non-tertiary	Higher	3
Qu 2008 [79]	Unclear	January 2006	December 2007	1	Retrospective	75	Multiple	Yes	Unclear	Unclear	Unclear	3
Qu 2011 [80]	Hubei	January 2006	January 2011	1	Retrospective	113	Multiple	No	Rural	Non-tertiary	Lower	4
Ren 2009 [81]	Ningxia	January 2007	August 2007	1	Retrospective	23	Multiple	Yes	Rural	Non-tertiary	Lower	2
Ruan 2011 [82]	Zhejiang	January 2007	December 2009	1	Retrospective	67	Abdominal	No	Urban	Tertiary	Higher	4
Sheng 2012 [83]	Zhejiang	January 2006	December 2011	1	Retrospective	41	Orthopedic	No	Rural	Tertiary	Higher	2
Shi 2011 [84]	Henan	January2006	December2009	1	Prospective	325	Multiple	Yes	Urban	Tertiary	Lower	5
Sun 2008 [85]	Shandong	March 2000	May 2005	1	Unclear	29	Ophthalmology	No	Urban	Tertiary	Higher	2
Sun 2012 [86]	Hubei	January 2009	December 2010	1	Retrospective	221	Multiple	Yes	Urban	Tertiary	Lower	2
Tang 2009 [87]	Hebei	January 2005	December 2005	Unclear	Unclear	300	Thoracic	No	Urban	Tertiary	Lower	2
Tang 2012 [88]	Guangdong	January 2008	December 2011	1	Retrospective	65	Unclear	No	Urban	Tertiary	Higher	3
Tao 2011 [89]	Hubei	January 2007	December 2009	1	Ambispective	20	Neurosurgery	No	Urban	Tertiary	Lower	3
Tian 2011 [90]	Hubei	January 2008	December 2009	1	Retrospective	Unclear	Multiple	Yes	Urban	Tertiary	Lower	3
Wan 2009 [91]	Hunan	January 2005	December 2008	3	Unclear	185	Multiple	No	Rural	Non-tertiary	Lower	3
Wang 2007a [92]	Beijing	January 2001	December 2005	1	Retrospective	48	Abdominal	Yes	Urban	Tertiary	Higher	2
Wang 2007b [93]	Hubei	Unclear	Unclear	Unclear	Unclear	Unclear	Unclear	Yes	Urban	Tertiary	Lower	1
Wang 2008 [94]	Hebei	January 2005	December 2007	1	Unclear	Unclear	Unclear	No	Urban	Tertiary	Lower	2
Wang 2012 [95]	Zhejiang	June 2003	June 2011	1	Retrospective	223	Abdominal	No	Urban	Tertiary	Higher	4
Wei 2010 [96]	Guangdong	January 2008	December 2008	1	Monitoring	53	Gynecologic	No	Urban	Tertiary	Higher	7
Xiang 2012 [97]	Zhejiang	March 2008	September 2009	1	Retrospective	47	Orthopedic	No	Rural	Non-tertiary	Higher	3
Xie 2007 [98]	Zhejiang	January 2003	December 2005	1	Prospective	211	Multiple	Yes	Urban	Tertiary	Higher	4
Xie 2008 [99]	Hubei	January 2004	December 2006	1	Retrospective	128	Multiple	Yes	Urban	Tertiary	Lower	4
Xie 2010 [100]	Hubei	November 2007	November 2008	10	Cross-sectional	80	Multiple	No	Urban	Tertiary	Lower	5
Xie 2012 [101]	Sichuan	January 2005	October 2011	1	Retrospective	426	Unclear	Yes	Urban	Tertiary	Lower	2
Xiu 2012 [102]	Heilongjiang	January 2011	December 2011	1	Retrospective	29	Multiple	No	Urban	Tertiary	Lower	4
Xu 2007 [103]	Beijing	August 1997	September 2006	1	Retrospective	Unclear	Neurosurgery	No	Urban	Tertiary	Higher	2
Xu 2010 [104]	Guangxi	Unclear	Unclear	Unclear	Prospective	124	Orthopedic	No	Urban	Tertiary	Lower	4
Xu 2011 [105]	Guangdong	November 2007	November 2010	1	Retrospective	31	Unclear	No	Urban	Non-tertiary	Higher	3
Yan 2008 [106]	Guangdong	January 2003	September 2006	1	Unclear	311	Multiple	No	Urban	Tertiary	Higher	4
Yang 2009a [107]	Sichuan	January 2006	December 2007	1	Retrospective	Unclear	Multiple	No	Rural	Non-tertiary	Lower	3
Yang 2009b [108]	Sichuan	January 2007	December 2007	1	Ambispective	46	Unclear	No	Urban	Non-tertiary	Lower	4
Yao 2011 [109]	Zhejiang	January 2005	December 2010	1	Retrospective	51	Abdominal	No	Urban	Non-tertiary	Higher	4
You 2011 [110]	Fujian	January 2001	June 2009	1	Retrospective	50	Thoracic	No	Urban	Tertiary	Higher	3
Yu 2012 [111]	Zhejiang	January 2009	July 2011	Unclear	Retrospective	398	Multiple	Yes	Urban	Non-tertiary	Higher	3
Yue 2009 [112]	Henan	June 2005	June 2008	1	Retrospective	21	Gynecologic	No	Rural	Non-tertiary	Lower	3
Zeng 2012 [113]	Hubei	January 2007	December 2010	Unclear	Retrospective	108	Gynecologic	Yes	Rural	Non-tertiary	Lower	2
Zhang 2007 [114]	Henan	January 2004	December 2005	1	Unclear	91	Orthopedic	No	Urban	Tertiary	Lower	3
Zhang 2008 [115]	Hubei	January 2004	December 2005	1	Unclear	145	Multiple	No	Urban	Tertiary	Lower	2
Zhang 2009a [116]	Sichuan	January 2009	June 2011	1	Retrospective	142	Unclear	No	Urban	Non-tertiary	Lower	3
Zhang 2009b [117]	Hubei	January 2006	December 2008	1	Retrospective	Unclear	Multiple	Yes	Urban	Tertiary	Lower	1
Zhang 2010 [118]	Hunan	January 2008	December 2008	1	Retrospective	47	Abdominal	No	Rural	Non-tertiary	Lower	4
Zhang 2011a [119]	Sichuan	January 2006	December 2008	1	Retrospective	160	Multiple	No	Urban	Non-tertiary	Lower	4
Zhang 2011b [120]	Zhejiang	January 2007	December 2009	1	Retrospective	87	Abdominal	No	Urban	Tertiary	Higher	2
Zhang 2011c [121]	Hainan	March 2007	March 2009	1	Retrospective	30	Multiple	No	Urban	Tertiary	Lower	3
Zhang 2012 [122]	Gansu	January 2008	December 2009	Unclear	Ambispective	252	Multiple	Yes	Urban	Tertiary	Lower	5
Zhao 2011a [123]	Guizhou	January 2008	December 2010	1	Retrospective	72	Multiple	No	Urban	Tertiary	Lower	3
Zhao 2011b [124]	Shandong	January 2008	December 2010	1	Retrospective	227	Multiple	Yes	Urban	Tertiary	Higher	4
Zheng 2007 [125]	Zhejiang	January 2005	December 2005	1	Retrospective	Unclear	Urologic	Yes	Urban	Tertiary	Higher	2
Zheng 2011a [126]	Hubei	January 2008	December 2009	Unclear	Unclear	148	Unclear	Yes	Urban	Tertiary	Lower	4
Zheng 2011b [127]	Hubei	January 2008	December 2009	1	Retrospective	87	Abdominal	Yes	Urban	Non-tertiary	Lower	5
Zheng 2011c [128]	Zhejiang	January 2005	December 2010	1	Cross-sectional	41	Multiple	No	Urban	Tertiary	Higher	4
Zhou 2007 [129]	Hainan	January 2001	December 2005	1	Retrospective	38	Gynecologic	No	Urban	Tertiary	Lower	2
Zhou 2008 [130]	Hainan	January 2001	December 2005	1	Retrospective	18	Gynecologic	No	Urban	Non-tertiary	Lower	3
Zhou 2011a [131]	Hubei	January 2005	December 2009	1	Retrospective	1172	Multiple	Yes	Urban	Tertiary	Lower	5
Zhou 2011b [132]	Beijing	October 2009	September 2011	1	Prospective	24	Thoracic	Yes	Urban	Tertiary	Higher	3
Zhu 2007 [133]	Hubei	January 2002	December 2006	1	Retrospective	138	Abdominal	No	Urban	Tertiary	Lower	3
Zhu 2008a [134]	Guizhou	January 2006	January 2008	1	Retrospective	63	Orthopedic	No	Urban	Non-tertiary	Lower	4
Zhu 2008b [135]	Jiangxi	January 2000	December 2006	1	Retrospective	Unclear	Multiple	Yes	Urban	Tertiary	Lower	4
Zhu 2010 [136]	Guangdong	December 2009	March 2010	1	Unclear	20	Gynecologic	No	Urban	Tertiary	Higher	3

* In this column multiple surgeries refer to the different kinds of surgeries involved in the study which cannot be discriminated or classified into a specific type of surgery.

**Table 2:** Distribution of *S. aureus* and MRSA isolates in the included studies.

**Characteristics**		***S. aureus***			**MRSA**		
		**Number of studies**	**Number of *S.aureus***	**Number of detected isolates**	**Number of studies**	**Number of MRSA**	**Number of *S. aureus***
Publication year	2007	13	351	2,441	4	43	95
	2008	16	380	1,636	6	84	198
	2009	16	285	1,806	3	40	123
	2010	14	200	1,257	4	42	92
	2011	24	714	3,613	11	221	539
	2012	23	582	2,855	10	194	455
Surgery type	Orthopedic	16	362	2,022	5	35	143
	Abdominal	15	161	1,163	3	35	64
	Gynecologic	13	88	471	2	10	25
	Thoracic	4	88	196	2	19	73
	Others*	49	1,556	8,754	23	445	1,003
	Unclear	8	257	1,029	3	80	194
Study design	Retrospective	67	1,794	10,169	26	520	1,204
	Prospective	10	139	781	4	34	95
	Ambispective	9	117	764	1	11	29
	Cross-sectional	3	38	133	1	1	2
	Unclear	17	424	1,761	6	58	172
Regions	Urban	85	2,111	11,603	31	563	1,344
	Rural	20	378	1,925	6	55	135
	Unclear	1	23	80	1	6	23
Hospitals	Tertiary	63	1,740	9,581	25	499	1,168
	Non-tertiary	42	749	3,947	12	119	311
	Unclear	1	23	80	1	6	23
Economic Condition	Higher	45	852	5,734	12	152	384
	Lower	60	1,637	7,794	26	466	1,095
	Unclear	1	23	80	1	6	23
Study Quality	Higher	39	1,000	5,313	14	271	665
	Lower	67	1,512	8,295	24	353	837
Sample Size**	>20 isolates	95	2,464	13,435	26	561	1,392
	≤20 isolates	11	48	173	12	63	110
Total		106	2,512	13,608	38	624	1,502

* Others refer to: 1) multiple surgeries involved in the study which cannot be classified into a specific type of surgery or 2) a specific type of surgery, rather than orthopedic, abdominal, gynecologic, or thoracic surgeries, which was reported in a small number of studies.

** Sample size refers to isolates of all identified bacteria for the proportion of *S.aureus*, isolates of all identified *S.aureus* for the proportion of MRSA, and isolates of MRSA for the proportion of antibiotic resistance.

### Methodological quality of studies

**Figure 2:**
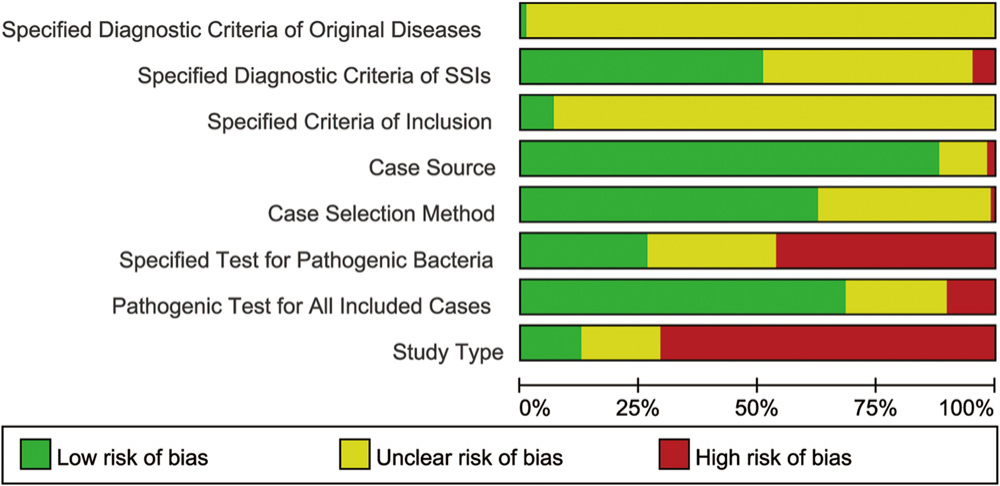
Summary of risk of bias for all the included studies.

The methodological quality of studies was displayed in Fig. 2 with more details in S2 Table, S1 Fig The maximum score that studies achieved was 7 while the minimum was 0. We finally identified 38 studies with relatively high quality which reached at least 4 scores in our quality assessment scale.

### Overall proportions

**Figure 3:**
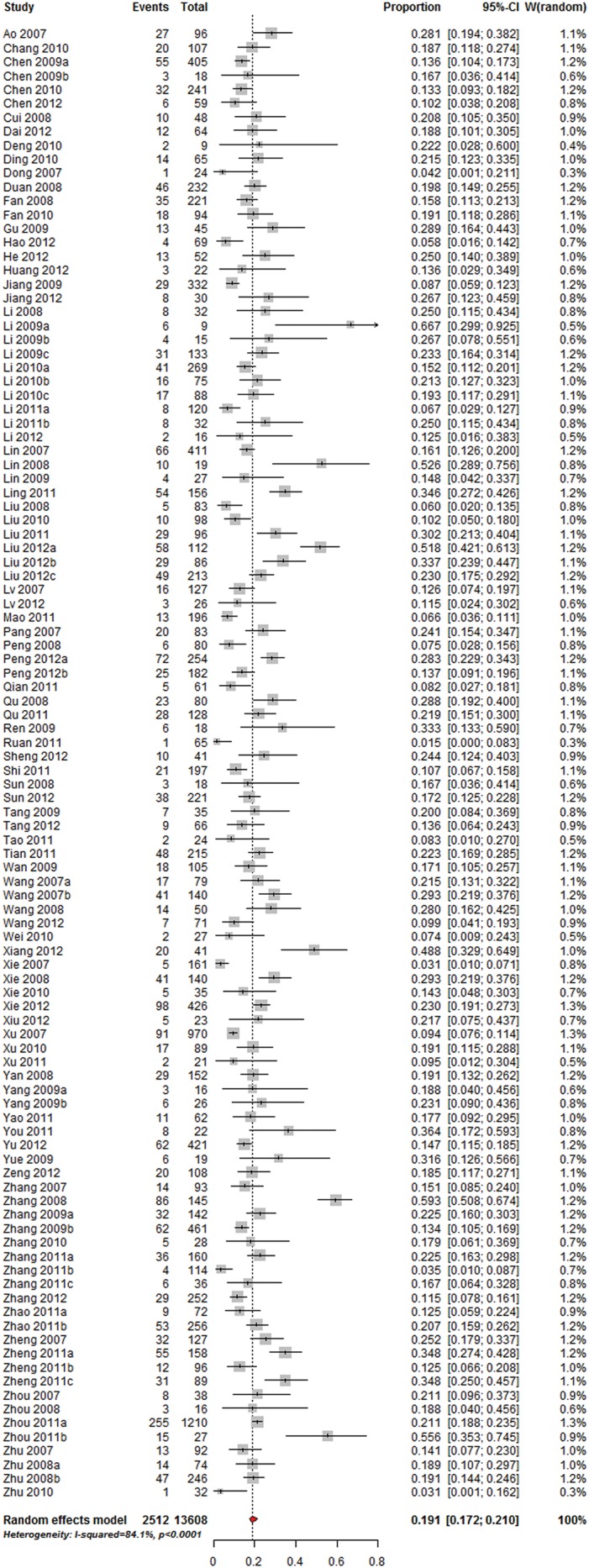
Overall proportion of *S. aureus* in patients with SSIs.

106 studies, including a total of 13,608 isolates, reported proportions of *S. aureus* isolates. The pooled proportion of *S. aureus* isolates among patients with SSIs was 19.1% (95%CI 17.2–21.0%; I^2^ = 84.1%) (Fig. 3). The proportion was similar to 18.5% (1,452/7,848, 95%CI 17.7–19.4%) (Q = 0.32, df = 1, P-value = 0.570) in the US, but significantly exceeded the proportions of 12.7% (377/2,971, 95%CI 11.5–13.9%) (Q = 33.4, df = 1, P-value<0.001) and 13.5% (515/3,812, 95%CI 12.5–14.6%) (Q = 28.3, df = 1, P-value<0.001) reported through the China Nosocomial Infection Surveillance System.

**Figure 4:**
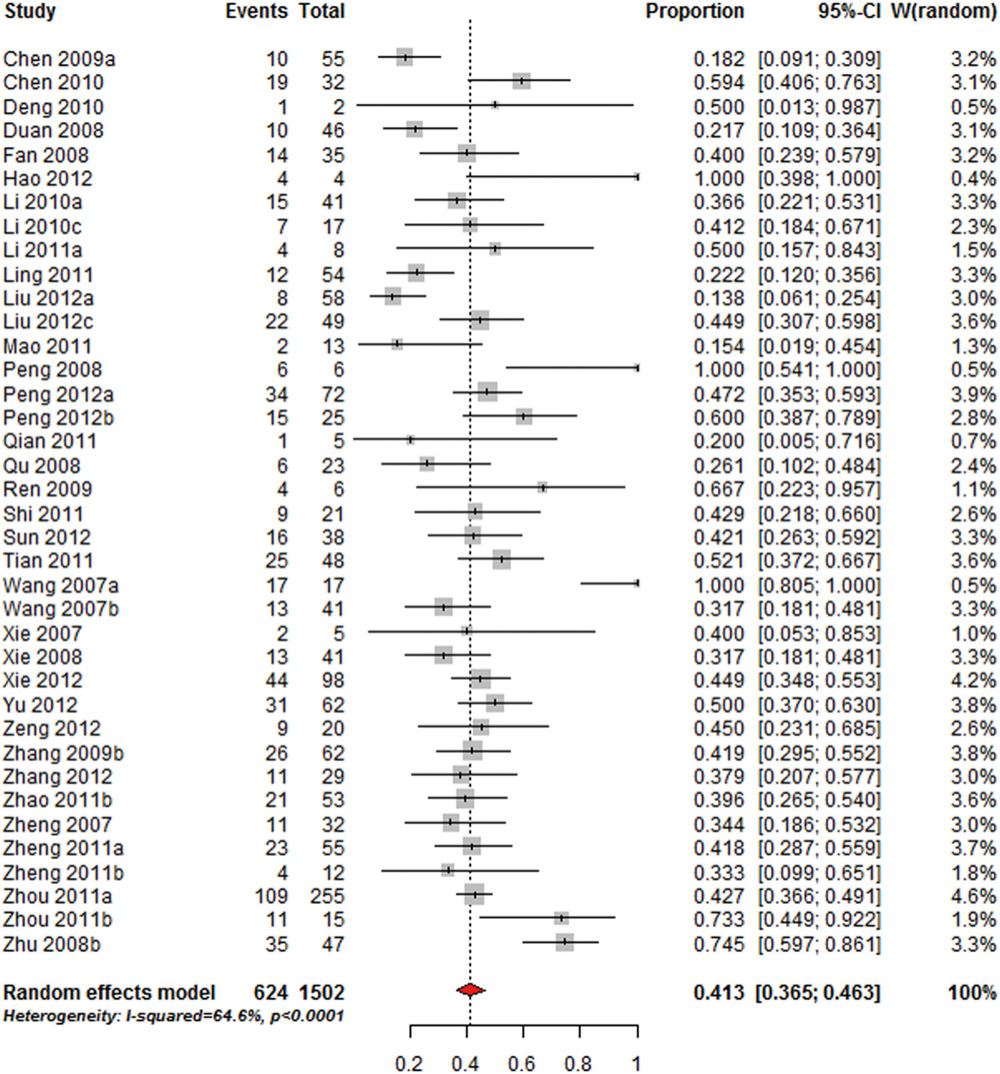
Overall proportion of MRSA in patients with *S. aureus* SSIs.

With respect to the proportion of MRSA, 1,502 isolates of *S. aureus* in 38 studies were included. The overall proportion of MRSA isolates was 41.3% (95%CI 36.5–46.3%; I^2^ = 64.6%) (Fig. 4). The proportion was significantly lower (Q = 10.3, df = 1, P-value = 0.001) than that of 53.9% in the US (150/278, 95%CI 48.1–59.7%).

No evidence in Egger’s test suggested publication bias for the overall proportion of *S. aureus* (t=-1.10, P-value = 0.275) and MRSA (t = 0.46, P-value = 0.651).

### Subgroup analysis

All the results of subgroup analysis were summarized in Table 3 and the forest plots were presented in S1 File.

**Table 3:** Summary of the pooled results of proportions of *S. aureus* and MRSA isolates.

**Subgroup**		**Proportions of *S. aureus* isolates**					**Proportions of MRSA isolates**				
		**Studies**	Sample Size**	**Estimate (%)**	**95%CI (%)**	**I^2^ (%)**	**Studies**	Sample Size**	**Estimate (%)**	**95%CI (%)**	**I^2^ (%)**
Surgery type	Orthopedic	16	2,022	20.4	15.3–26.7	87.8	5	143	26.6	15.3–42.2	56.9
	Abdominal	15	1,281	13.8	10.3–18.4	70.0	3	64	55.0	21.4–84.5	74.1
	Gynecologic	13	471	20.1	15.6–25.6	33.0	2	25	41.0	23.5–61.1	0
	Thoracic	4	196	41.1	26.3–57.7	74.4	2	73	39.1	3.8–91.2	94.0
	Others*	50	8,609	18.2	15.9–20.7	85.6	23	1,003	44.6	39.5–49.7	51.5
	Unclear	8	1,029	24.7	20.1–30.0	60.2	3	194	41.3	34.4–48.6	3.0
Economic condition	Higher	45	5,777	16.6	13.9–19.7	84.2	12	384	39.4	28.6–51.2	73.4
	Lower	60	7,751	20.7	18.5–23.2	81.8	25	1,095	42.8	37.5–48.3	59.4
	Unclear	1	80	28.8	19.9–39.6	-	1	23	26.1	12.2–47.2	-
Region	Urban	85	11,678	18.5	16.4–20.7	85.8	31	1,344	41.5	36.3–46.9	66.5
	Rural	20	1,850	20.9	17.4–25.0	69.4	6	135	44.9	29.2–61.7	60.7
	Unclear	1	80	28.8	19.9–39.6	-	1	23	26.1	12.2–47.2	-
Hospital Level	Tertiary	63	9,613	18.3	15.9–21.0	88.2	25	1,168	42.7	36.9–48.7	69.1
	Non-tertiary	42	3,915	20.0	17.5–22.6	68.8	12	311	39.0	30.0–48.7	52.2
	Unclear	1	80	28.8	19.9–39.6	-	1	23	26.1	12.2–47.2	-
Quality	Higher	39	5,225	17.9	15.5–20.6	78.5	14	665	40.1	32.6–48.0	65.6
	Lower	67	8,383	19.9	17.4–22.8	86.3	24	837	42.1	35.7–48.9	65.3
Study Design	Retrospective	67	10,169	18.6	16.9–20.5	75.6	26	1,204	42.4	37.2–47.7	62.2
	Non-retrospective**	22	1,678	17.9	13.1–23.8	84.4	6	126	41.3	26.3–58.0	59.4
	Unclear	17	1,669	21.6	14.9–30.2	92.1	6	172	41.0	22.9–61.9	75.5
Sample size***	>20 isolates	95	13,435	18.6	16.7–20.5	85.2	26	1,392	39.8	35.0–44.8	68.2
	≤20 isolates	11	173	28.1	19.8–38.1	38.7	12	110	53.6	36.1–70.3	53.1
Start time	Before 2007	55	8,696	18.8	16.4–21.4	85.2	17	821	41.0	33.4–49.0	72.2
	Since 2007	47	4,642	19.0	16.1–22.3	83.2	19	638	42.3	35.6–49.2	60.1
	Unclear	4	270	25.5	20.6–31.1	0.1	2	43	32.6	20.3–47.8	0
Total		106	13,608	19.1	17.2–21.0	84.1	38	1,502	41.3	36.5–46.3	64.6

* Others refer to: 1) multiple surgeries involved in the study which cannot be classified into a specific type of surgery or 2) a specific type of surgery, rather than orthopedic, abdominal, gynecologic, or thoracic surgeries, which was reported in a small number of studies.

** Non-retrospective design comprises prospective, cross-sectional, ambispective study and surveillance.

*** Sample size in the proportions of *S. aureus* isolates refers to the number of all the detected bacteria isolates; in the proportions of MRSA it refers to the number of all *S. aureus* isolates.

The pooled proportion was 41.1% (95%CI 26.3–57.7%; I^2^ = 74.4%) for thoracic surgeries, 20.4% (95%CI 15.3–26.7%; I^2^ = 87.8%) for orthopedics surgeries, 20.1% (95%CI 26.3–57.7%; I^2^ = 74.4%) for gynecologic surgeries and 13.8% (95%CI 10.3–18.4%; I^2^ = 70.0%) for abdominal surgeries. In addition, *S. aureus* proportion was higher in studies conducted in low economic condition, rural or non-tertiary hospitals or with small sample size (at most 20 isolates of bacteria), although significant differences between subgroups were not found. On the other hand, the proportions seemed similar in studies with high and low quality, those with retrospective and non-retrospective design, or those beginning before and since 2007 (Fig. A-I in S1 File).

**Figure 5:**
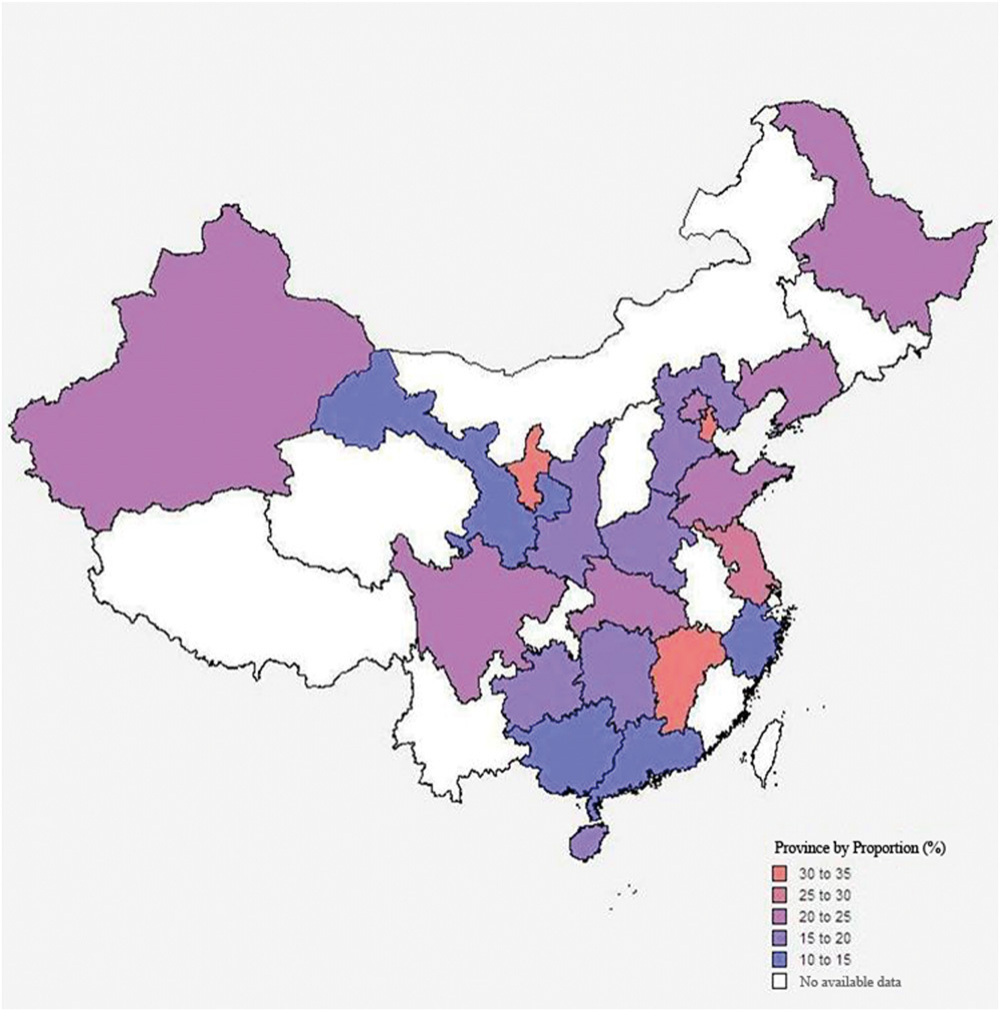
Province distribution of proportions of *S. aureus* isolates in China.

Geographical differences in *S. aureus* proportions by different provinces or municipalities across China were shown in Fig. 5. Among 21 areas the maximum point estimate of *S. aureus* proportion among all the provinces with available data was 33.3% (95%CI 15.8–57.1%) in Ningxia province, followed by Tianjin municipality (30.2%, 95%CI 21.9–40.1%) and Jiangxi province (30.0%, 95%CI 16.9–47.4%) and the minimum was 11.5% (95%CI 8.1–16.1%) in Gansu province. However, there was only one study available, respectively, for the proportion estimate in Ningxia, Jiangxi and Gansu.

Regarding the pooled proportion of MRSA isolates (Fig. J-R in S1 File), the proportion was 55.0% (95%CI 21.4–84.5%, I^2^ = 74.1%) for abdominal surgeries, 41.0% (95%CI 23.5–61.1%, I^2^ = 0%) for gynecologic surgeries, 39.1% (95%CI 3.8–91.2%, I^2^ = 94.0%) for thoracic surgeries and 26.6% (95%CI 15.3–42.2%, I^2^ = 56.9%) for orthopedics surgeries. Furthermore, despite insignificant difference between subgroups, MRSA proportion tended to be higher in low economic condition, urban and tertiary hospitals as well as in studies with small sample size (at most 20 *S. aureus* isolates). Similar proportions can be found between studies with higher and lower quality, studies with retrospective and non-retrospective design, or studies with the start time of before 2007 and since 2007.

All the MRSA were sensitive to vancomycin (522/522) while only one isolate was resistant to linezolid (1/94). 79.9% (95%CI 67.4–88.4%; I^2^ = 0%) and 92.0% (95%CI 80.2–97.0%; I^2^ = 0%) of MRSA, respectively pooled from four and five studies, were resistant to clindamycin and erythromycin (Fig. S and Fig. T in S1 File).

### Meta-regression for the proportion of *S. aureus* isolates

97 studies without any missing data were included for meta-regression to identify related potential factors for heterogeneity with statistical significance. As we found a significant correlation coefficient between levels of hospital and region (coefficient = 0.570, P-value<0.001), provincial economic condition and region (coefficient=-0.198, P-value = 0.052), the region variable was therefore excluded out of the pre-defined independent factors for the meta-regression (Table 4).

**Table 4:** Summary results of meta-regression for the proportion of *S. aureus* isolates.

**Factor**		**Coefficient**	**SE**	**OR**	**95% CI (OR)**		**P-Value**
Surgery type							
	Thoracic	-	-	1	-	-	-
	Abdominal	-1.495	0.385	0.224	0.105	0.477	<0.001
	Gynecologic	-1.370	0.408	0.254	0.114	0.565	<0.001
	Orthopedic	1.043	0.369	0.352	0.171	0.726	0.005
	Others	-1.281	0.351	0.278	0.140	0.552	<0.001
Economic condition							
	Lower	-	-	1	-	-	-
	Higher	-0.232	0.139	0.793	0.604	1.041	0.095
Hospital level							
	Non-tertiary	-	-	1	-	-	-
	Tertiary	-0.223	0.143	0.800	0.605	1.058	0.118
Sample size							
	<20 isolates	-	-	1	-	-	-
	≥20 isolates	-0.541	0.268	0.582	0.344	0.985	0.044
Quality							
	Lower	-	-	1	-	-	-
	Higher	-0.080	0.141	0.923	0.700	1.218	0.573
Constant		-0.513	0.443	-	-	-	0.247

The meta-regression (residual I^2^ = 83.3%, adjusted R^2^ = 17.6%, P-value<0.001 in the test for the goodness of model fit) showed that compared with thoracic, *S. aureus* proportion was significantly lower in abdominal (OR = 0.224, 95%CI 0.105–0.477, P-value<0.001), gynecologic (OR = 0.254, 95%CI 0.114–0.565, P-value<0.001) and orthopedic (OR = 0.352, 95%CI 0.171–0.726, P-value = 0.005) surgeries. Studies with relatively large sample size (>20 isolates) were likely to conclude lower proportions of *S. aureus* (OR = 0.582, 95%CI 0.344–0.985, P-value = 0.044).

## Discussion

### Proportions of *S. aureus*

The overall proportion of *S. aureus* isolates (19.1%) was consistent with the reported proportion of 18.5% in the US in 2003 [21], but was significantly higher than both estimated in the China Nosocomial Infection Surveillance System (12.7% between 1999 and 2001, 13.5% between 1999 and 2005) [11, 12]. This difference between our review and the Chinese surveillance system could not be attributed to the change over time because of insignificant difference between the studies starting before 2007 and those starting after 2007 by subgroup analysis. However, the following reasons may result in such difference. First, the surveillance result derived from only 79 of 134 surveillance hospitals (58.9%), which may reduce the representativeness of the practical situation in China. Nevertheless, 106 studies in our review involved more than 125 hospitals distributed in 21 different provinces or municipalities, including tertiary and non-tertiary hospitals, urban and rural areas, which may be more representative of the real national status. Second, the sample size of bacteria isolates from the surveillance system (only 3,812 in total) was substantially less than that included in our review (13,608). Pooling data from 106 studies with a larger sample size may provide a more reliable estimate for national situation.

In addition, our finding can provide further useful information which was not available from the Chinese surveillance system, such as the stratified proportions by the surgery type, economics condition, hospital level and province. As shown in the subgroup analysis, *S. aureus* proportions varied between different surgery types—highest for thoracic surgeries (41.1%) whereas lowest for abdominal surgeries (13.8%). Meta-regression suggested the similar result that patients undergoing thoracic surgeries were much more vulnerable to SSIs by *S. aureus*, compared with patients with any other involved surgery type. This result was consistent with the guideline for prevention of SSIs, which concluded that *S. aureus* was the dominant pathogen causing SSIs following thoracic surgeries [8], indicating that *S. aureus* should be highly suspected in the case of SSIs after thoracic surgeries. On the contrary, with other influencing factors adjusted, patients with abdominal surgeries were less likely to suffer from SSIs by *S. aureus*. Priority may not be given to *S. aureus* in the SSIs after this surgery type because gram-negative bacilli, rather than *S. aureus*, tend to be predominant in the gastrointestinal tract usually involved in abdominal surgeries [22, 23]. Impoverished regions, non-tertiary hospitals, and some provinces or municipalities such as Ningxia, Tianjin and Jiangxi, may also require more attention paid to *S. aureus* SSIs.

### Proportions of MRSA

Our estimate focused on SSIs and thus may close the gap of the surveillance system in China which merely reported the MRSA proportion of 79.9% (3,177/3,975) in all kinds of hospital infections instead of in SSIs [12]. Comparison between the results indicated that the proportion of MRSA in SSIs may be lower than the average level among a diversity of hospital infections. Besides, our review concluded a significantly lower proportion than that reported in a recent multi-center study with a smaller sample size in the US [24]. However, the status quo necessitates further improvement since MRSA accounted for more than 40% of *S. aureus* in our review.

Variation in the MRSA proportions was found between different surgery types: highest in abdominal surgeries (55.0%) and lowest in orthopedics surgeries (26.6%). While a recent study showed cases with MRSA SSIs accounted for 30.4% in those with *S. aureus* SSIs in the US [22], the high MRSA proportion (55.0%) following abdominal surgeries in our study provided an alarming picture that, despite *S. aureus* being subordinate pathogen in SSIs after abdominal surgeries, physicians still have to be highly cautious about MRSA in SSIs. On the contrary, orthopedic surgeries saw the lowest proportion of MRSA SSIs (26.6%) in spite of its high proportion of *S. aureus*. A study also concluded that the proportion of MRSA was the lowest in orthopedic surgeries among all the surgical procedures, although the proportion (31.9%) they calculated was higher than ours [10]. However, the mechanism seems still unclear and requires further studies to confirm.

### Proportions of antibiotic-resistant MRSA

Based on our findings, vancomycin and linezolid appeared to be still effective for treating MRSA in SSIs in vitro. Vancomycin therapy is the primary option in the case of limited current therapeutic methods for patients with MRSA infections [25]. In China, the surveillance system suggested that none of MRSA were resistant to vancomycin (0/3,102) between 1999 and 2005 in a variety of nosocomial infections including SSIs [12], which was similar to our result: we identified none of the MRSA isolates resistant to vancomycin (0/522) in SSIs. However, it is necessary to raise the awareness of the resistance of vancomycin since there has been evidence suggesting the observed rise in minimum inhibitory concentrations (MICs) of vancomycin from less than 0.5μg/mL in 2005 to 1.0 μg/mL in 2010 [26]. Linezolid is the first available oxazolidinone antibiotic, which uniquely inhibits bacterial protein synthesis by preventing formation of 70S initiation complex [27]. Although surveillance data on linezolid was absent, one of 94 MRSA isolates in our findings was resistant to linezolid. But currently no robust clinical evidence can demonstrate whether linezolid or vancomycin is superior in the treatment of MRSA SSIs [28]. Continuous surveillance of drug resistance for both antibiotics in this treatment is necessary and crucial for the clinical practice.

On the other hand, clindamycin and erythromycin, inhibiting protein synthesis by their effect on ribosome function and commonly used in clinical practice for MRSA SSIs [27, 29], may have a doubtful effectiveness. The proportion of MRSA resistant to clindamycin in our findings (79.9%) was also similarly suggested in the surveillance system for nosocomial infections—78.9% (1,137/1,445) [12]. In our review, more than 90.0% MRSA isolates were identified to be resistant to erythromycin, far higher than that in the UK bacteraemia surveillance where erythromycin resistance only occurred in 67% of MRSA [30]. As such, both treatments may not be the first choice when MRSA in SSIs is suspected.

### Limitations

There are several limitations in this review. First, methodological quality of the included studies is the main concern for the combined estimates because less than half of the studies are of high quality according to our criteria. However, study quality seems not to be the main heterogeneity source as both subgroup analysis and meta-regression showed that the pooled result from studies of higher quality were consistent with that from those of lower quality. Second, we only included studies published after 2007 so as to understand the current proportion of *S. aureus* and MRSA in SSIs. Considering some studies had started before 2007, we conducted subgroup analysis for studies initiating before and after 2007 to ensure that it was reasonable to combine results from all included studies to provide more precise estimates and facilitate the meta-regression. Third, none of the pre-defined variables can fully explain the variance in proportions of *S. aureus* (I^2^ = 84.1%) and MRSA (I^2^ = 64.6%) in subgroup analysis and meta-regression, which could result in uncertainty around the pooled proportions. The major obstacle of extensively exploring the potential source of variation is the limited information about the heterogeneity reported in the publication, such as the duration of surveillance, MICs and molecular epidemiology, which may be significantly associated with the heterogeneity but cannot be examined in our review. However, meta-regression did find that some factors with available information, such as the types of surgery and sample sizes, may partly contribute to the heterogeneity across studies. In addition, despite informal comparisons between subgroups by 95%CI rather than the significance test, the problem with multiple comparisons may be raised in the comparisons with no adjustment made with a stricter criterion for the significant difference. Further study may also be required to confirm some pooled results derived from limited number of included studies in our review.

## Conclusion

In conclusion, the overall proportion of *S. aureus* causing SSIs in mainland China was similar to that in the US, and the proportion of MRSA was possibly lower. The real proportion of *S. aureus* may be higher than that reported from the Chinese surveillance system. Both proportions of *S. aureus* and MRSA tended to depend on types of surgeries. Therefore, clinicians should take into account the types of surgery when taking care of post-operative patients and managing *S. aureus* and MRSA SSIs. Vancomycin and linezolid appeared to be effective for MRSA in SSIs. Further well-designed studies on this topic, including surveillance and primary prospective studies, are required to provide further reliable evidence.

## Supporting Information

S1 Fig Risk of bias for each included study. (TIF)

S1 File Subgroup analyses. (DOCX)

S1 PRISMA Checklist (DOCX)

S1 Table Search strategies and results. (DOCX)

S2 Table Quality assessment of the included studies. (DOCX)

S3 Table Distribution of MRSA isolates resistant to specific antibiotics. (DOCX)

## References

[1] Horan TC, Gaynes RP, Martone WJ, Jarvis WR, Emori TG (1992) CDC definitions of nosocomial surgical site infections, 1992: a modification of CDC definitions of surgical wound infections. Am J Infect Control 20: 271–274. 10.1016/S0196-6553(05)80201-9 1332552

[2] Coello R, Charlett A, Wilson J, Ward V, Pearson A, et al (2005) Adverse impact of surgical site infections in English hospitals. Journal of Hospital Infection 60: 93–103. 10.1016/j.jhin.2004.10.019 15866006

[3] Anderson D, Kaye K (2009) Staphylococcal surgical site infections. Infect Dis Clin North Am 23: 53–72. 10.1016/j.idc.2008.10.004 19135916

[4] Astagneau P, Rioux C, Golliot F, Brücker G, INCISO Network Study Group (2001) Morbidity and mortality associated with surgical site infections: results from the 1997–1999 INCISO surveillance. J Hosp Infect 48: 267–274. 10.1053/jhin.2001.1003 11461127

[5] Smyth E, McIlvenny G, Enstone J, Emmerson A, Humphreys H, et al (2008) Four country healthcare associated infection prevalence survey 2006: overview of the results. J Hosp Infect 69: 230–248. 10.1016/j.jhin.2008.04.020 18550218

[6] Martone W, Nichols R (2001) Recognition, prevention, surveillance, and management of surgical site infections: introduction to the problem and symposium overview. Clin Infect Dis 33: S67–68. 10.1086/321859 11486301

[7] de Lissovoy G, Fraeman K, Hutchins V, Murphy D, Song D, et al (2009) Surgical site infection: incidence and impact on hospital utilization and treatment costs. Am J Infect Control 37: 387–397. 10.1016/j.ajic.2008.12.010 19398246

[8] Mangram AJ, Horan TC, Pearson ML, Silver LC, Jarvis WR (1999) Guideline for prevention of surgical site infection, 1999. American Journal of Infection Control 27: 97–134. 10.1016/S0196-6553(99)70088-X 10196487

[9] Jernigan J (2004) Is the burden of Staphylococcus aureus among patients with surgical-site infections growing? Infect Control Hosp Epidemiol 25: 457–460. 10.1086/502421 15242191

[10] Engemann JJ, Carmeli Y, Cosgrove SE, Fowler VG, Bronstein MZ, et al (2003) Adverse clinical and economic outcomes attributable to methicillin resistance among patients with Staphylococcus aureus surgical site infection. Clin Infect Dis 36: 592–598. 10.1086/367653 12594640

[11] Wu A, Ren N, Wen X, Xu X, Li J (2005) Pathogens of surgical site infection. Zhonghua Yi Yuan Gan Ran Xue Za Zhi 15: 210–212.

[12] Ren N, Wen X, Wu A (2007) Study on surveillance of drug resistance of Staphylococcus aureus in hospital infection in National Monitoring Net of Hospital Infection. Zhongguo Yi Xue Gong Cheng 5: 425–427.

[13] Wen X, Ren N, Wu A, Xu X (2011) Distribution of pathogens causing nosocomial infection monitored by national nosocomial infection surveillance system and changing trend. Zhonghua Yi Yuan Gan Ran Za Zhi 21: 350–355.

[14] Wu A, Wen X, Ren N, Xu X, Yi X, et al (2005) Pathogens causing nosocomial infection monitored by national nosocomial infection surveillance system and their change. Proceedings of the Twelfth Annual Academic Conference of the National Hospital Infection Management.

[15] National Collaborating Centre for Acute Care (2003) Preoperative tests: the use of routine preoperative tests for elective surgery. National Institute for Health and Clinical Excellence. 21089235

[16] Borenstein M, Hedges L, Higgins J, Rothstein H (2009) Introduction to Meta-analysis. Chichester (UK): John Wiley & Sons.

[17] Higgins J, Thompson S, Deeks J, Altman D (2003) Measuring inconsistency in meta-analyses. BMJ 327: 557–560. 10.1136/bmj.327.7414.557 12958120 PMC192859

[18] National Bureau of Statistics of China (2011) China Statistical Yearbook-2011. Beijing: China Statistics Press.

[19] Deeks J, Higgins J, Altman D (2011) Chapter 9: Analysing data and undertaking meta-analyses. In: Higgins J, Green S, editors. Cochrane Handbook for Systematic Reviews of Interventions Version 510 [updated March 2011]: The Cochrane Collaboration.

[20] Egger M, Davey S, Schneider M, Minder C (1997) Bias in meta-analysis detected by a simple, graphical test. BMJ 315: 629–634. 10.1136/bmj.315.7109.629 9310563 PMC2127453

[21] Gaynes R, Edwards J, National Nosocomial Infections Surveillance System (2005) Overview of nosocomial infections caused by gram-negative bacilli. Clin Infect Dis 41: 848–854. 10.1086/432803 16107985

[22] Ramirez MC, Marchessault M, Govednik-Horny C, Jupiter D, Papaconstantinou HT (2012) The Impact of MRSA Colonization on Surgical Site Infection Following Major Gastrointestinal Surgery. J Gastrointest Surg: 1–9. 10.1007/s11605-012-1995-2 22948833

[23] Tlaskalová-Hogenová H, Stepánková R, Hudcovic T, Tucková L, Cukrowska B, et al (2004) Commensal bacteria (normal microflora), mucosal immunity and chronic inflammatory and autoimmune diseases. Immunol Lett 93: 97–108. 10.1016/j.imlet.2004.02.005 15158604

[24] Anderson D, Kaye K, Chen L, Schmader K, Choi Y, et al (2009) Clinical and financial outcomes due to methicillin resistant Staphylococcus aureus surgical site infection: a multi-center matched outcomes study. PLoS One 4: e8305 10.1371/journal.pone.0008305 20016850 PMC2788700

[25] Stevens D, Herr D, Lampiris H, Hunt J, Batts D, et al (2002) Linezolid versus vancomycin for the treatment of methicillin-resistant Staphylococcus aureus infections. Clin Infect Dis 34: 1481–1490. 10.1086/340353 12015695

[26] Zhao C, Sun H, Wang H, Liu Y, Hu B, et al (2012) Antimicrobial resistance trends among 5608 clinical Gram-positive isolates in China: results from the Gram-Positive Cocci Resistance Surveillance program (2005–2010). Diagn Microbiol Infect Dis 73: 174–181. 10.1016/j.diagmicrobio.2012.03.003 22521693

[27] Santayana E, Jourjy J (2011) Treatment of methicillin-resistant Staphylococcus aureus surgical site infections. AACN Adv Crit Care 22: 5–12. 10.1097/NCI.0b013e3181ef86fe2049019 21297385

[28] Gurusamy K, Koti R, Toon C, Wilson P, Davidson B (2013) Antibiotic therapy for the treatment of methicillin-resistant Staphylococcus aureus (MRSA) infections in surgical wounds. Cochrane Database Syst Rev 8: CD009726 23963687 10.1002/14651858.CD009726.pub2 PMC11301404

[29] Weisblum B (1995) Erythromycin resistance by ribosome modification. Antimicrob Agents Chemother 39: 577–585. 10.1128/AAC.39.3.577 7793855 PMC162587

[30] Gemmell CG, Edwards DI, Fraise AP, Gould FK, Ridgway GL, et al (2006) Guidelines for the prophylaxis and treatment of methicillin-resistant Staphylococcus aureus (MRSA) infections in the UK. J Antimicrob Chemother 57: 589–608. 10.1093/jac/dkl017 16507559

[31] Ao F, Wang B, Peng X, Liu X, Wang J, et al (2007) Distribution and Drug Resistance of Pathogens from incisional infections after orthopedic surgery [in Chinese]. Gannan Yi Xue Yuan Xue Bao 27: 883–884, 896.

[32] Chang F (2010) General Surgery-related Factors of Incision Infection and Interventions [in Chinese]. Zhonghua Yi Yuan Gan Ran Xue Za Zhi 20: 1674–1676.

[33] Chen B (2009) The analysis of the bacteria of the incisional infections in orthopedics operation in recent 3 years [in Chinese]. Chinese Nursing Care Association Conference of National Hospital Infections. pp. 50–54.

[34] Chen H, Yang X, Fu X, Chen H, Deng Y (2009) Prospective study of the correlation between the time of draingage tube intubation after open cranial operation and the incidence of lacunar infection of intracranial organ [in Chinese]. Xian Dai Yi Yuan 9: 11–12.

[35] Chen L, Zheng Y, Ma D (2010) Drug-resistance of Pathogens Isolated from Operative Incision [in Chinese]. Zhonghua Yi Yuan Gan Ran Xue Za Zhi 20: 419–420.

[36] Chen X, Lin D, Lin H (2012) The surgical site infections of the pathogen research hospitals [in Chinese]. Zhongguo Yi Yao Zhi Nan 10: 4–5.

[37] Cui Q, Li X, Ji S (2008) Investigation on incisional infection of 1001 cases after surgical operation in 2007 [in Chinese]. Yu Fang Yi Xue Lun Tan 14: 989–990.

[38] Dai C (2012) Risk factors for incisional infections due to colorectal cancer surgery [in Chinese]. Zhonghua Yi Yuan Gan Ran Xue Za Zhi 22: 3532–3533.

[39] Deng X, Zhang L, Liu Z, Ma L, Dou F (2010) Cross-sectional survey on surgical site infections in 10 hospitals [in Chinese]. Zhonghua Yi Yuan Gan Ran Xue Za Zhi 20: 1672–1673, 1682.

[40] Ding H (2010) Investigation of the incisional infections in surgery [in Chinese]. Liaoning Yi Xue Za Zhi 24: 3.

[41] Dong Y (2007) The status and influence factors of patients’ infections after surgery [in Chinese]. Zhejiang: Zhejiang University 51 p.

[42] Duan Q, Chen H, Liu C (2008) The monitoring of bacterial drug resistance of incisional infections in the primary hospital [in Chinese]. Zhongguo Shi Yan Zhen Duan Xue 12: 1468–1469.

[43] Fan W, Huang E, Duan L (2008) Distribution and drug resistance of pathogens in abdominal surgical wounds [in Chinese]. Zhonghua Yi Yuan Gan Ran Xue Za Zhi 18: 1562–1563.

[44] Fan T, Hu H, Sun J (2010) Clinical observation of 36 cases with limb injury and incisional infections [in Chinese]. Zhongguo Yi Shi Jin Xiu Za Zhi 33: 30–32.

[45] Gu S, Mai W (2009) The analysis of 76 cases with incisional infectons on abdomen after caesarean section [in Chinese]. Zhongguo She Qu Yi Shi:.

[46] Hao M (2012) The anlysis and investigation of the bacteria in and after open fracture surgery [in Chinese]: Hebei Medical University. 32 p.

[47] He Y (2012) The analysis and countermeasures of incisional infections in aseptic operations [in Chinese]. Nanchang Da Xue Xue Bao 52: 69–71.

[48] Huang Y, Li L (2012) The anlysis of incisional infections after colorectal surgery (19 cases) [in Chinese]. Zhongguo Yi Yao Zhi Nan 10: 143–144.

[49] Jiang Y, Kan Y, Wei W, Wei X (2009) The anlysis and preventive strategy of the bacteria in wound infections after open fractures surgery [in Chinese]. Zhejiang Lin Chuang Yi Xue 11: 390–392.

[50] Jiang Y (2012) Targeted monitoring of incisional infections caused by cesarean section [in Chinese]. Zhonghua Yi Yuan Gan Ran Xue Za Zhi 22: 1391–1392.

[51] Li D (2008) The clinical analysis of abdominal incisional infections after gynecologic surgery in 36 cases [in Chinese]. Zhongguo Shi Yong Yi Yao 3: 58–59.

[52] Li D, Yang Y, Su H (2009) The surveillance of surgical site infections in a hospital in Neijiang [in Chinese]. Luzhou Yi Xue Yuan Xue Bao 32: 396–398.

[53] Li L, Lin Z (2009) Clinical analysis of 15 cases with abdominal incisional infectons after caesarean section [in Chinese]. Zhongguo Dang Dai Yi Yao 16: 162–163.

[54] Li M, Wei R (2009) An investigation of incisional infections after orthopedic aseptic operations in 14274 cases [in Chinese]. Zhong Yi Zheng Gu 21: 22–24.

[55] Li Y (2010) Drug-resistance of pathogens from infected surgical wounds [in Chinese]. Zhonghua Yi Yuan Gan Ran Xue Za Zhi 20: 2338–2339.

[56] Li Z, Shi W, Tan J (2010) Investigation on distribution and drug resistance of pathogenic bacteria in surgical incisional infections [in Chinese]. Shi Yong Yu Fang Yi Xue 17: 1430–1432.

[57] Li X, Qin J, Feng Z, Gao W, Wang P (2010) Risk factors for surgical site infections in open fractures [in Chinese]. Zhonghua Yi Yuan Gan Ran Xue Za Zhi 20: 773–774.

[58] Li H, Wei Y, Zheng P, Ling P, Li J (2011) Drug resistance of pathogens causing nosocomial infections [in Chinese]. Zhonghua Yi Yuan Gan Ran Xue Za Zhi 21: 2122–2123.

[59] Li Y (2011) Clinic research about the cure of incision infection after gynecologic surgery [in Chinese]. An Mo Yu Kang Fu Yi Xue: 126–127.

[60] Li X, Yang W (2012) The investigation and countermeasure of incisional infections after obstetric operations [in Chinese]. Zhongguo She Qu Yi Shi 14: 300.

[61] Lin Z (2007) Analysis on common pathogens of surgical site infections and their drug resistance [in Chinese]. Zhi Ye Yu Jian Kang 23: 72–73.

[62] Lin X, Zhan Y, Cai X (2008) Perioperative nursing care of aseptic incisional infections after orthopedics opertaions [in Chinese]. Guangdong Yi Xue 29: 1598–1599.

[63] Lin S, Wu Y, Qiu L (2009) Investigation and analysis on risk factors for incisional wound infection after abdominal operations [in Chinese]. Yi Yao Shi Jie 11: 485–486.

[64] Ling L, Zhang D (2011) Bacterial culture from wound secretion and drug susceptibility [in Chinese]. Zhonghua Yi Yuan Gan Ran Xue Za Zhi 21: 3044–3046.

[65] Liu L, Wei Q, Zhang H, Zhong F, Su W (2008) Incisional infections after abdominal operations: investigation and strategy [in Chinese]. Zhonghua Yi Yuan Gan Ran Xue Za Zhi 18: 1091–1092.

[66] Liu C, Cui X (2010) Analysis of incisional infections in 274 surgical cases [in Chinese]. Yi Yao Lun Tan Za Zhi 31: 116–117.

[67] Liu W, Liu Y, Zhang N, Yan G (2011) Clinical analysis of orthopaedic sterile surgical incision infection and nursing countermeasures [in Chinese]. Zhonghua Yi Yuan Gan Ran Xue Za Zhi 21: 2689–2690.

[68] Liu C, Zhao H, Hu J, Wen Y, Yang X (2012) The distribution and drug resistance of the pathogens in incisional infections after surgical treatment of breast cancer [in Chinese]. Jian Yan Yi Xue Yu Lin Chuang 9: 2167–2168.

[69] Liu Z (2012) The analysis of 128 cases with incisional infections after abdominal operations [in Chinese]. Zhongguo Wei Sheng Chan Ye 9: 143–144.

[70] Liu X, Zhang C (2012) Drug resistance of pathogenic bacteria causing surgical site infections and intervention countermeasures [in Chinese]. Zhonghua Yi Yuan Gan Ran Xue Za Zhi 22: 4385–4386.

[71] Lv X, Huang J, Zhou P (2007) The study of distribution and antibacterial agents resistance of microorganism in general surgical site infections [in Chinese]. Zhongguo Wei Sheng Tai Xue Za Zhi 19: 213–214.

[72] Lv S, Qu J, Xiang Y, Yang K, Jin C, et al (2012) Pathogens causing surgical incisional infections afer colorectal cancer surgery and related factors [in Chinese]. Zhonghua Yi Yuan Gan Ran Xue Za Zhi 22: 4507–4508.

[73] Mao G, Sun G (2011) Distribution and drug resistance of pathogens in patients with surgical incisional infections in orthotedics department [in Chinese]. Zhongguo Wei Sheng Jian Yan Za Zhi 21: 910–912.

[74] Pang J (2007) The analysis of surgical incisional infections and antibiotic resistance [in Chinese]. Xi Bu Yi Xue 19: 85–86.

[75] Peng L, Chen X (2008) The analysis of the incisional infections in rural hospitals [in Chinese]. The Fifth Academic Conference of Analytic Microbiology Professional Committee of Hubei Microbiology Association. pp. 415–417.

[76] Peng Y (2012) Distribution and risk factors of pathogenic bacteria causing surgical incisional infections [in Chinese]. Zhonghua Yi Yuan Gan Ran Xue Za Zhi 22: 840–842.

[77] Peng Y, Chen X (2012) The analysis of drug resistance of the pathogens in surgical wound infections [in Chinese]. Zhongguo Wu Zhen Xue Za Zhi 12: 1618–1619.

[78] Qian P, Li J, Ye J (2011) Pathogens causing perineal incisional infections and prevention measures [in Chinese]. Zhonghua Yi Yuan Gan Ran Xue Za Zhi 21: 819–820.

[79] Qu L, Zhang X (2008) The analysis of pathogenic bacteria in surgical incisional infections in primary level hospital [in Chinese]. The Third Academic Conference of National Bacteria Resistance Surveillance and Clinical Speciality pp. 519–520.

[80] Qu C (2011) Investigation and intervention of incisional infections after surgeries [in Chinese]. Zhongguo Wei Sheng Chan Ye 8: 78.

[81] Ren G, Hu F (2009) Pathogens distribution and drug sensitivity of the incisional infections: analysis of 23 cases [in Chinese]. Zhongguo She Qu Yi Shi 11: 181.

[82] Ruan Y, Qin B, Guo L (2011) Factors associated with surgical site infections in patients with colorectal cancer [in Chinese]. Zhonghua Yi Yuan Gan Ran Xue Za Zhi 21: 2691–2693.

[83] Sheng F (2012) Clinical characteristics of incisional infections after orthopedic surgeries and prevention measures [in Chinese]. Zhonghua Yi Yuan Gan Ran Xue Za Zhi 22: 4513–4514.

[84] Shi W, Shi W, Li H (2011) Prospective surveillance on surgical site infections [in Chinese]. Zhongguo Gan Ran Kong zhi Za Zhi 10: 123–125.

[85] Sun S, Liu M, Yang X (2008) Analysis of pathogens in postoperative infections on eyes [in Chinese]. Zhongguo Bing Yuan Sheng Wu Xue Za Zhi 3: 136–138.

[86] Sun G, Shi L (2012) Distribution of pathogens causing surgical site infections and analysis of antimicrobial resistance [in Chinese]. Zhonghua Yi Yuan Gan Ran Xue Za Zhi 22: 843–844.

[87] Tang M, Cui L, Shi D, Liang Y, Ma Q, et al (2009) Central venous catheter related infections and risk factors after cardiovascular surgery [in Chinese]. Shan Xi Yi Xue Za Zhi 38: 47–49, 76.

[88] Tang X, Xiao X, Chen S, Huang L, Tian W (2012) The cause of incisional infections, pathogens distribution and their drug sensitivity in 65 cases [in Chinese]. Guangdong Yi Xue Yuan Xue Bao 30: 411–412.

[89] Tao G (2011) Neurological surgical site infections and countermeasures [in Chinese]. Zhonghua Yi Yuan Gan Ran Xue Za Zhi 21: 1544–1545.

[90] Tian L, Shen J (2011) Investigation on antimicrobial resistance of pathogens from surgical incisions [in Chinese]. Lin Chuang Xue Ye Xue Za Zhi 24: 83–85.

[91] Wan W, He Z, Zhang R, Wang J (2009) Distribution of pathogenic bacteria in surgical incisional infections and analysis of their antibiotics resistance [in Chinese]. Zhongguo Xian Dai Yi Sheng 47: 68–69, 71.

[92] Wang A, Zhou J, Ma X, Dong L, Li G (2007) Surgical site infections after pancreas surgery and the use of perioperative antibiotics [in Chinese]. Zhongguo Yi Xue Ke Xue Yuan Xue Bao 29: 566–570. 19209807

[93] Wang L (2007) The analysis of distribution and drug resistance of the pathogens in incisional infections [in Chinese]. Academic Conference of Environment and Health.

[94] Wang Y, Wu D, Wang C (2008) The investigation about pathogens in open wounds in different time of doctors’ office visiting [in Chinese]. Zhonghua Yi Yuan Gan Ran Xue Za Zhi 18: 1783.

[95] Wang X, Zhang Q, Chen J, Luo J (2012) Monitoring and analysis of postoperatice incisional infections in patients with gastrointestinal cancer [in Chinese]. Zhonghua Yi Yuan Gan Ran Xue Za Zhi 22: 2322–2324.

[96] Wei L, Wen J, Li Z, Qiu Y (2010) Analysis on targeted surveillance data of incisional infections after cesarean section [in Chinese]. Zhongguo Fu You Bao Jian 25: 3395–3398.

[97] Xiang D, Lian Y (2012) Analysis of risk factors and control measures for orthopedic sterile surgical wound infections [in Chinese]. Zhonghua Yi Yuan Gan Ran Xue Za Zhi 22: 1150–1152.

[98] Xie D, Xu X, Wang S (2007) The analysis of bacteria with extended spectrum beta-lactamases in incisional infections [in Chinese]. Xian Dai Zhong Xi Yi Jie He Za Zhi 16: 5121–5122.

[99] Xie M, Yan W, Xiong J (2008) Pathogen distribution and drug resistance in postoperative incisional infections [in Chinese]. Zhonghua Yi Yuan Gan Ran Xue Za Zhi 18: 874–876.

[100] Xie DS, Xiong W, Xiang LL, Fu XY, Yu YH, et al (2010) Point prevalence surveys of healthcare-associated infection in 13 hospitals in Hubei Province, China, 2007–2008. J Hosp Infect 76: 150–155. 10.1016/j.jhin.2010.04.003 20692727

[101] Xie X, Yang C, Bi J, Chen W, Zhou J (2012) Pathogens causing surgical site infections and drug resistance [in Chinese]. Zhonghua Yi Yuan Gan Ran Xue Za Zhi 22: 4387–4389.

[102] Xiu Y, He T, Wang Y, Yao Y (2012) Analysis on drug-resistance pathogens in Class Ⅰ surgical wound infections [in Chinese]. Hu Li Yan Jiu 26: 2631–2633.

[103] Xu M, Shi Z, Tang M, Zhou J (2007) Distribution and antimicrobial resistance of bacteria isolated from cerebral spinal fluid in neurosurgical patients: a ten years surveillance [in Chinese]. Beijing Yi Xue 29: 583–586.

[104] Xu X, Huang Z, Liu J (2010) Distribution and drug resistance of bacteria isolated from postoperative infections in orthopedic patients [in Chinese]. Zhonghua Yi Yuan Gan Ran Xue Za Zhi 20: 1304–1306.

[105] Xu Y (2011) The causative analysis on incisional infections after abdominal operation in 31 cases [in Chinese]. Zhong Wai Jian Kang Wen Zhai 8: 181–182.

[106] Yan B, Li M, Xu X (2008) The distribution and antimicrobial resistance of pathogenic oganism in surgical site Infections [in Chinese]. Jiangxi Yi Xue Yuan Xue Bao 48: 49–51, 55.

[107] Yang C (2009) Surgical incisional infections in a rural hospital in the impoverished area [in Chinese]. Zhonghua Yi Yuan Gan Ran Xue Za Zhi 19: 1512–1513.

[108] Yang X, Zhang Y, Guo T, Li X (2009) Monitoring survey about the incisional infections after surgery in 46 cases [in Chinese]. Xian Dai Yi Yao Wei Sheng 25: 1676–1678.

[109] Yao D (2011) Distribution of pathogens from infected surgical incisions in colorectal cancer and its influencing factors [in Chinese]. Zhongguo Xian Dai Yi Sheng 49: 147–148, 154.

[110] You H, Liao C, Yang X, Shan Z, Zhao X, et al (2011) Risk factors of surgical site infections in adult patients after cardiopulmonary by-pass surgery [in Chinese]. Zhonghua Yi Yuan Gan Ran Xue Za Zhi 21: 894–896.

[111] Yu Y, Chen Z (2012) Hospital surgical site infections: distribution of pathogens and detection of drug resistance [in Chinese]. Zhonghua Yi Yuan Gan Ran Xue Za Zhi 22: 1282–1284.

[112] Yue X (2009) The clinical analysis on incisional infections after gynecologic surgery [in Chinese]. Zhongguo Shi Yong Yi Yao 4: 183.

[113] Zeng L, Zhang C (2012) Drug resistance in pathogens causing surgical incisional infections after cesarean section and prevention measures [in Chinese]. Zhonghua Yi Yuan Gan Ran Xue Za Zhi 22: 1285–1286.

[114] Zhang R, Yang Y (2007) Etiology and drug sensitivity analysis of wound infections in the department of orthopaedics [in Chinese]. Guo Ji Jian Yan Yi Xue Za Zhi 28: 25–27.

[115] Zhang Z, Cao Y (2008) Study on the isolation and drug resistance of common pathogenic bacteria in general surgery infections [in Chinese]. The Fifth Academic Conference of Analytic Microbiology Professional Committee of Hubei Microbiology Association. pp. 220–225.

[116] Zhang X (2009) An analysis of the antimicrobial-resistant pathogens in surgical incisional intections [in Chinese]. Zhonghua Shi Yong Zhong Xi Yi Za Zhi 22: 1491–1492, 1494.

[117] Zhang Y, Lv W (2009) Hospital postoperative infections: distribution of pathogens and detection of drug resistance [in Chinese]. Zhongguo Wei Sheng Jian Yan Za Zhi 19: 1315–1317.

[118] Zhang X, Luo L (2010) Investigation and prevention of the risk factors of incision infection after abdominal operation [in Chinese]. Nanhua Da Xue Xue Bao 38: 301–302.

[119] Zhang X, Peng F (2011) An investigation of the pathogens distribution and their drug resistance in 160 cases with incisional infections after a general surgery [in Chinese]. Yi Yao Qian Yan 1: 47.

[120] Zhang Y, Qian X (2011) Incisional infections after abdominal operations: investigation and analysis [in Chinese]. Zhonghua Yi Yuan Gan Ran Xue Za Zhi 21: 695–696.

[121] Zhang Y (2011) The related factors and the prevention of incisional infections in primary level hospital [in Chinese]. Xian Dai Yu Fang Yi Xue 38: 590–591.

[122] Zhang J, Zhang J (2012) The distribution and drug resistance of the pathogens in incisional infections [in Chinese]. Zhongguo Lin Chuang Yan Jiu 25: 338–339.

[123] Zhao W (2011) Investigation and analysis of incisional infection in general surgery department [in Chinese]. Zhonghua Yi Yuan Gan Ran Xue Za Zhi 21: 3849–3850.

[124] Zhao X, Liang Y, Wang W, Zhang J (2011) Drug-resistance of pathogens isolated from operative incisions [in Chinese]. Zhongguo Shi Yong Yi Kan 38: 27–28.

[125] Zheng S (2007) Characteristics and prevention of the incisional infections in urologic surgical procedures [in Chinese]. Xian Dai Yu Fang Yi Xue 34: 3799–3800.

[126] Zheng H, Xie M (2011) Antimicrobial resistance of pathogens causing surgical site infections in suizhou and interventions [in Chinese]. Zhonghua Yi Yuan Gan Ran Xue Za Zhi 21: 601–602.

[127] Zheng H (2011) Risk factors for abdominal surgical wound infections and pathogenic bacteria investigation [in Chinese]. Zhonghua Yi Yuan Gan Ran Xue Za Zhi 21: 270–271.

[128] Zheng S, Peng G, Xie L, Zhang C (2011) Pathogens causing wound infections after pediatric surgery and drug resistance [in Chinese]. Zhonghua Yi Yuan Gan Ran Xue Za Zhi 21: 5310–5312.

[129] Zhou B, Li C (2007) Distribution and drug resistance of pathogenic bacteria in incisional wounds in puerperae [in Chinese]. Zhongguo Re Dai Yi Xue 7: 2144–2146.

[130] Zhou B, Li C (2008) Clinical analysis of 18 cases with abdominal incisional infectons after cesarean section [in Chinese]. Shi Yong Fu Chan Ke Za Zhi 24: 436–437.

[131] Zhou J, Wen X (2011) Distribution and drug resistance of the pathogens in incisional infections after surgery in Shiyan area [in Chinese]. Jian Yan Yi Xue Yu Lin Chuang 8: 972–974.

[132] Zhou Y, Tian P, Du W, Rong Y (2011) Effect of linezolid for the Gram-positive cocci infections in sternum osteomyelitis [in Chinese]. Zhonghua Sun Shang Yu Xiu Fu Za Zhi 6: 50–51.

[133] Zhu J, Tong H (2007) Investigation of surgical site infections after abdominal surgery in our hospital in recent 5 years [in Chinese]. Ji Ceng Yi Xue Lun Tan 11: 485–486.

[134] Zhu Y, Wang L (2008) Distribution and drug resistance of pathogenic bacteria in surgical wounds of orthopedic surgery [in Chinese]. Zhongguo Xian Dai Yi Yao Za Zhi 10: 121–122.

[135] Zhu Z, Zhu P, Shao J, Li H, Mao S (2008) Detection of pathogens and antimicrobial resistance in surgical incisional infections [in Chinese]. Zhonghua Yi Yuan Gan Ran Xue Za Zhi 18: 1186–1188.

[136] Zhu H, Ke Y, Lin X, Zhou N (2010) Infection outbreak caused by mycobacterim abscess after cesarean section [in Chinese]. Zhongguo Gan Ran Kong Zhi Za Zhi 9: 393–395.

