# Supplementary figures and images for: Proportions of Staphylococcus aureus and Methicillin-Resistant Staphylococcus aureus in Patients with Surgical Site Infections in Mainland China: A Systematic Review and Meta-Analysis
Source: PLoS One. 2015 Jan 20;10(1):e0116079. doi: 10.1371/journal.pone.0116079 (PMC4300093; doi:10.1371/journal.pone.0116079)

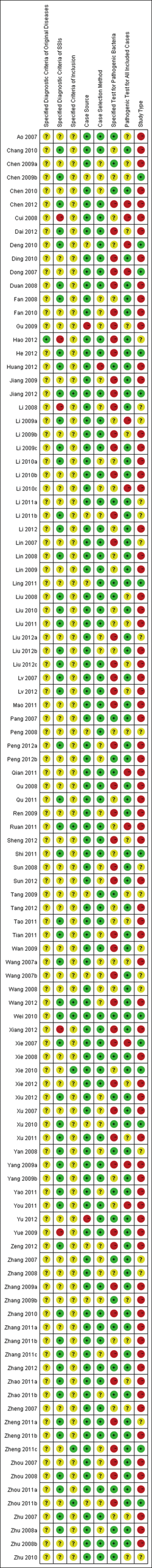

Supplement: S1 Fig — (TIF) [file pone.0116079.s001.tif]
