# Supplementary material for: Proportions of Staphylococcus aureus and Methicillin-Resistant Staphylococcus aureus in Patients with Surgical Site Infections in Mainland China: A Systematic Review and Meta-Analysis
Source: PLoS One. 2015 Jan 20;10(1):e0116079. doi: 10.1371/journal.pone.0116079 (PMC4300093; doi:10.1371/journal.pone.0116079)
Supplement: S1 File — (DOCX) [file pone.0116079.s002.docx]

**File S1. Subgroup analyses**

**
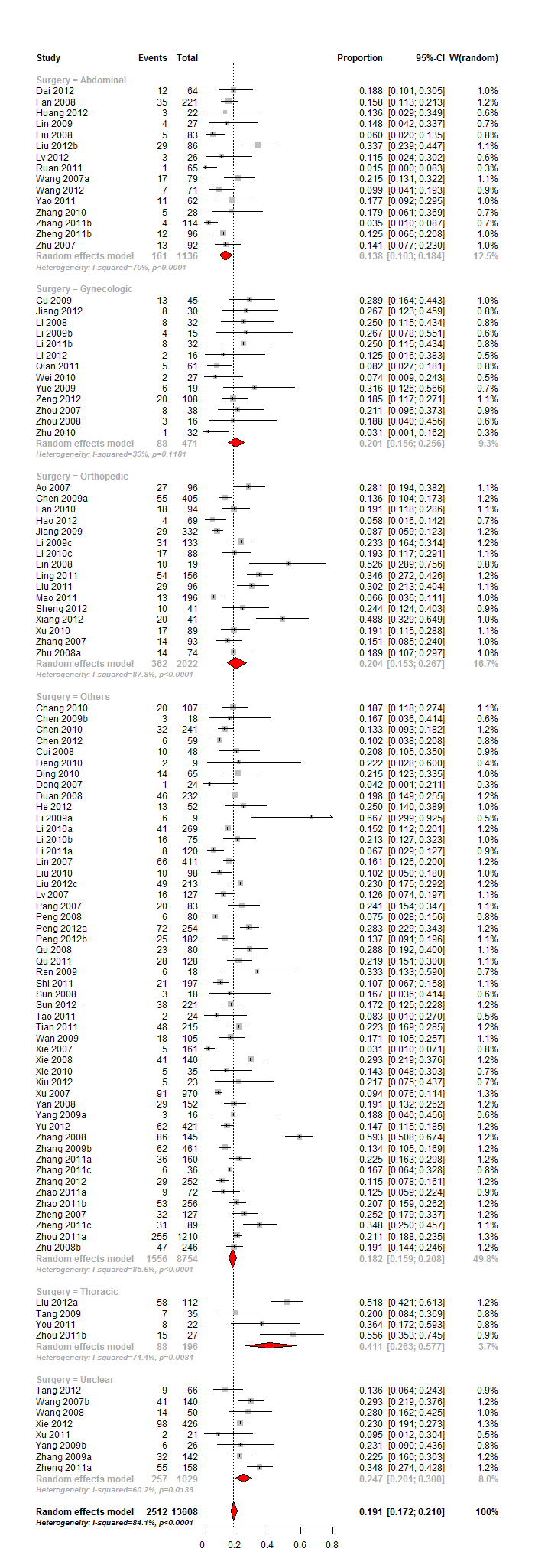
**

**Figure A. Subgroup analysis for the proportion of *S. aureus* by surgery type**


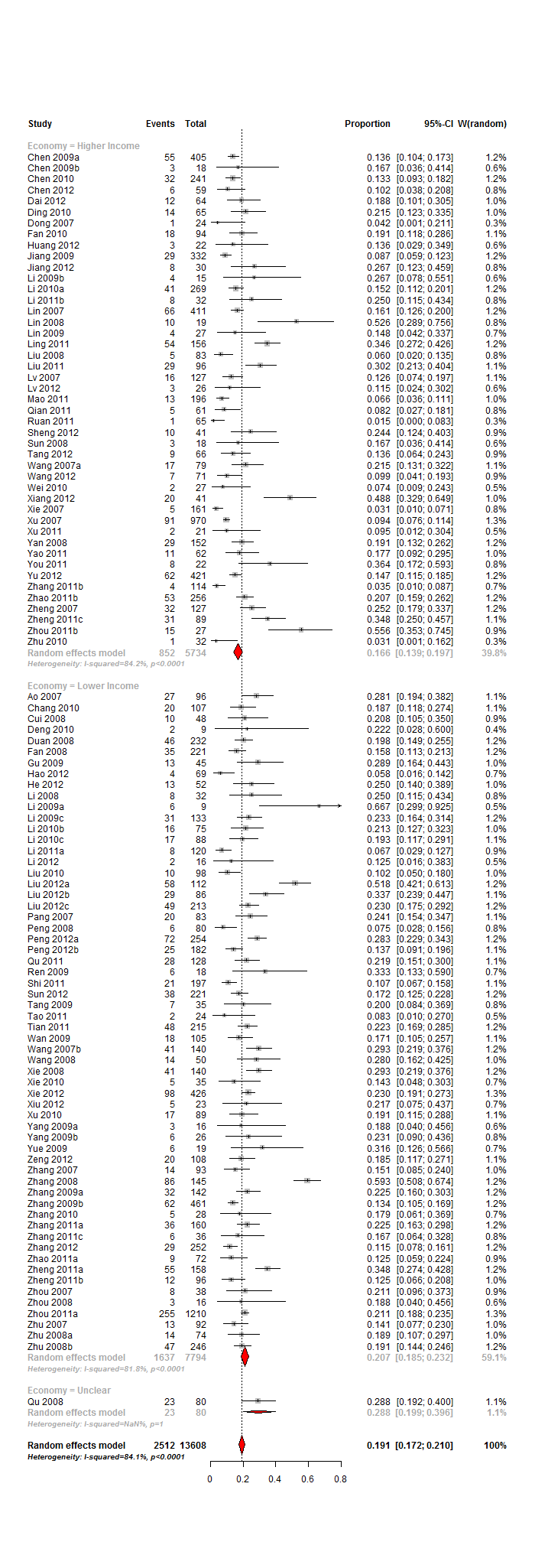


**Figure B. Subgroup analysis for the proportion of *S. aureus* by economic condition**


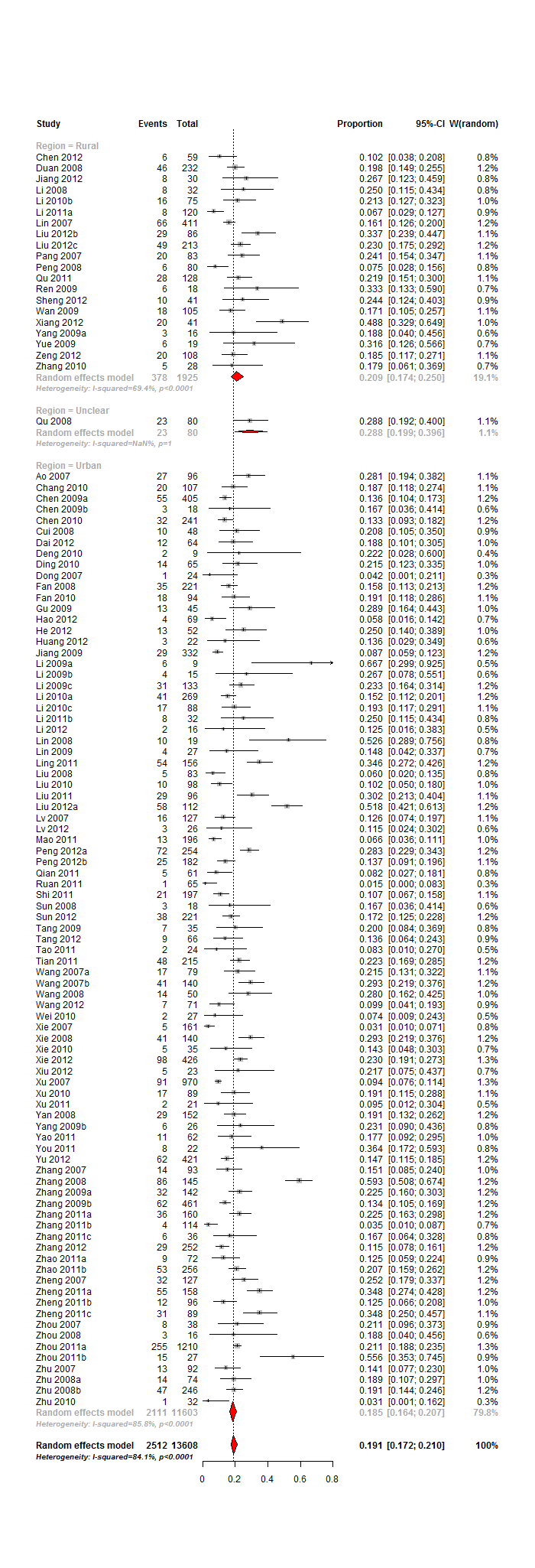


**Figure C. Subgroup analysis for the proportion of *S. aureus* by region**


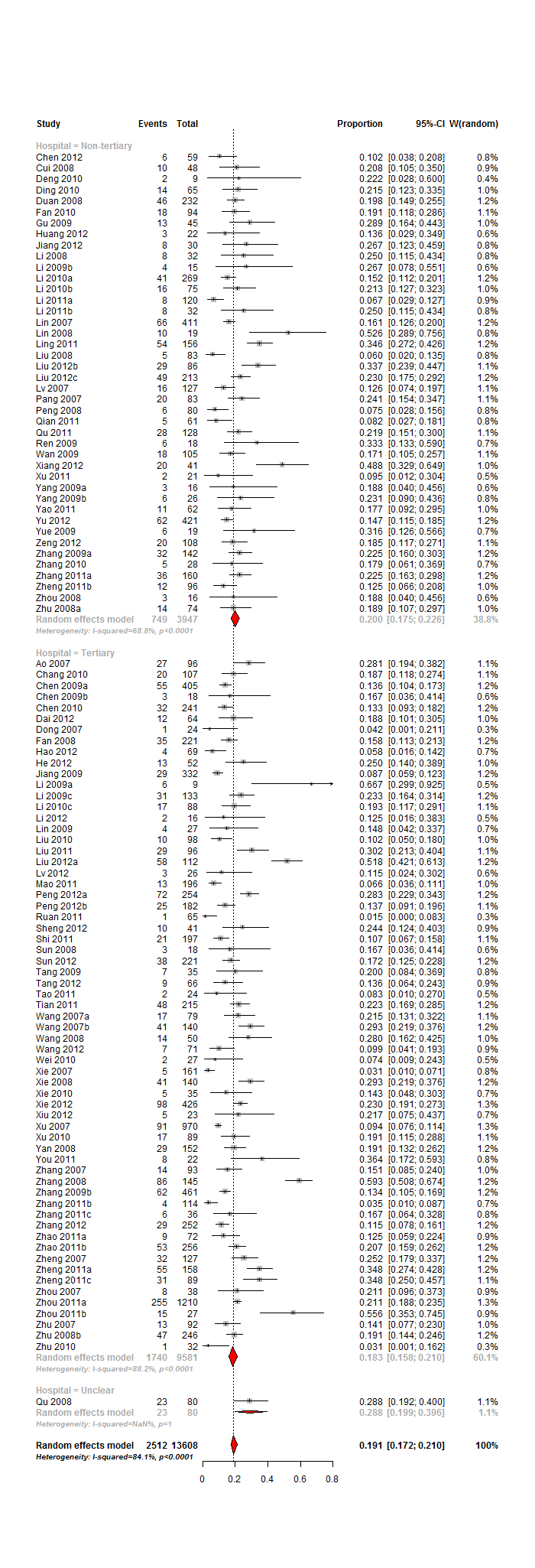


**Figure D. Subgroup analysis for the proportion of *S. aureus* by hospital level**


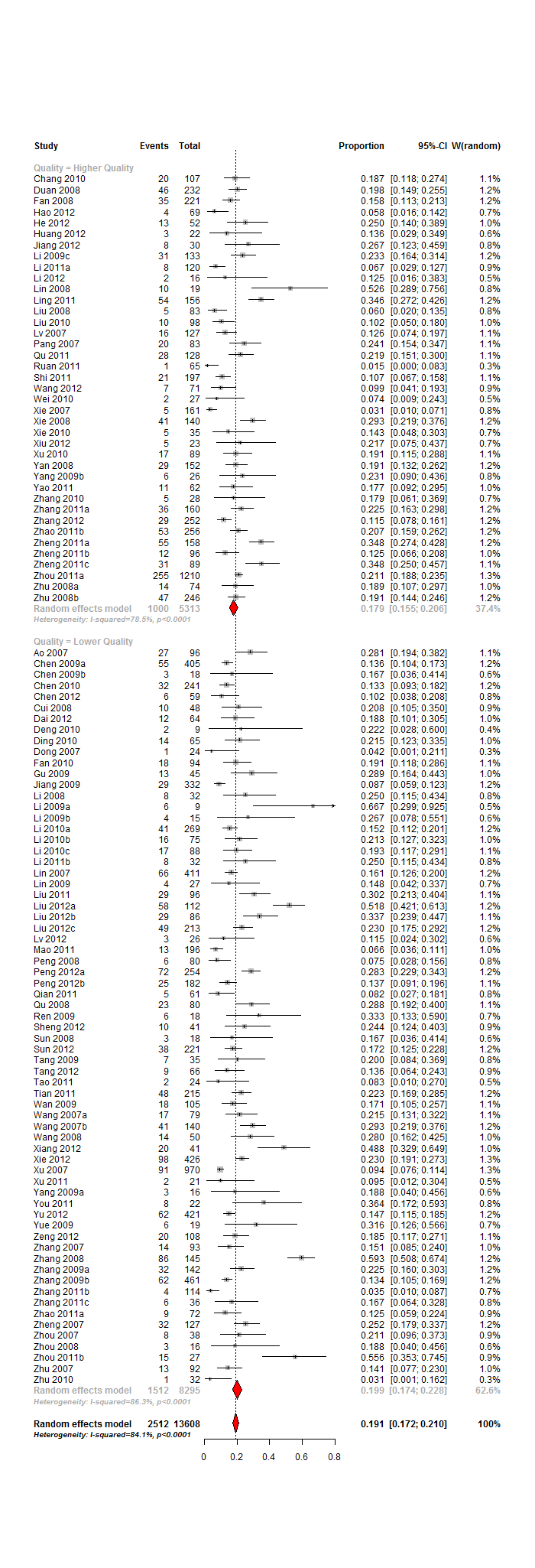


**Figure E. Subgroup analysis for the proportion of *S. aureus* by study quality**


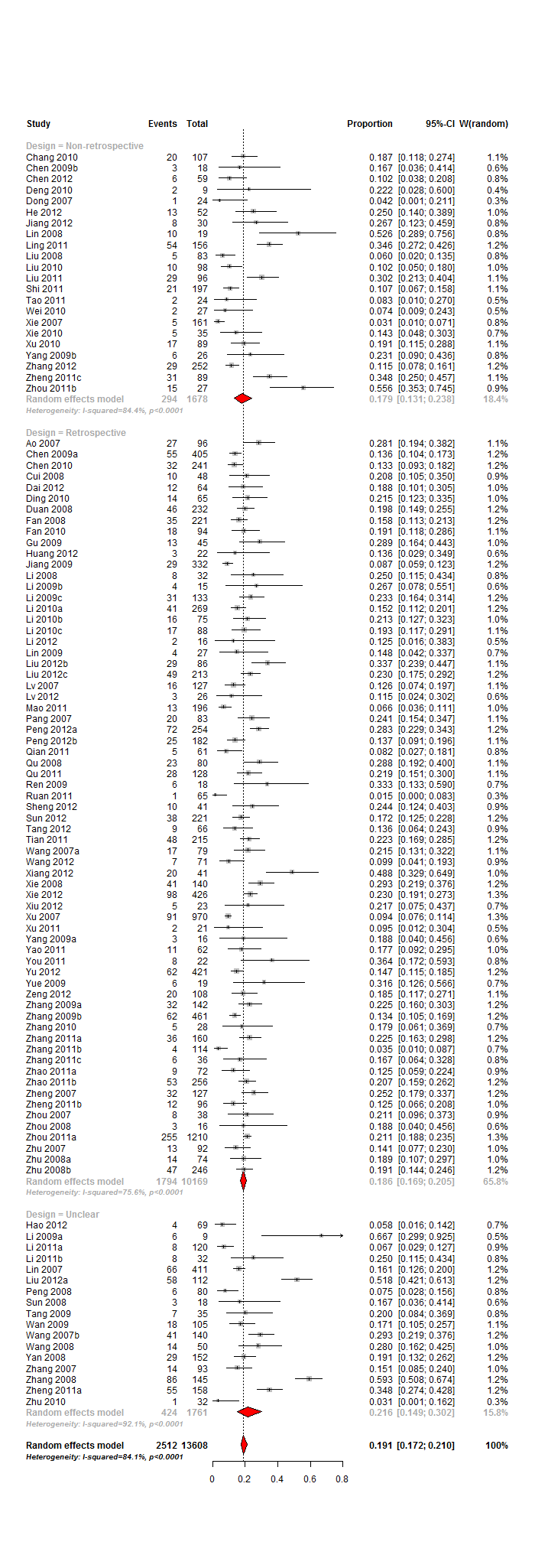


**Figure F. Subgroup analysis for the proportion of *S. aureus* by study design**


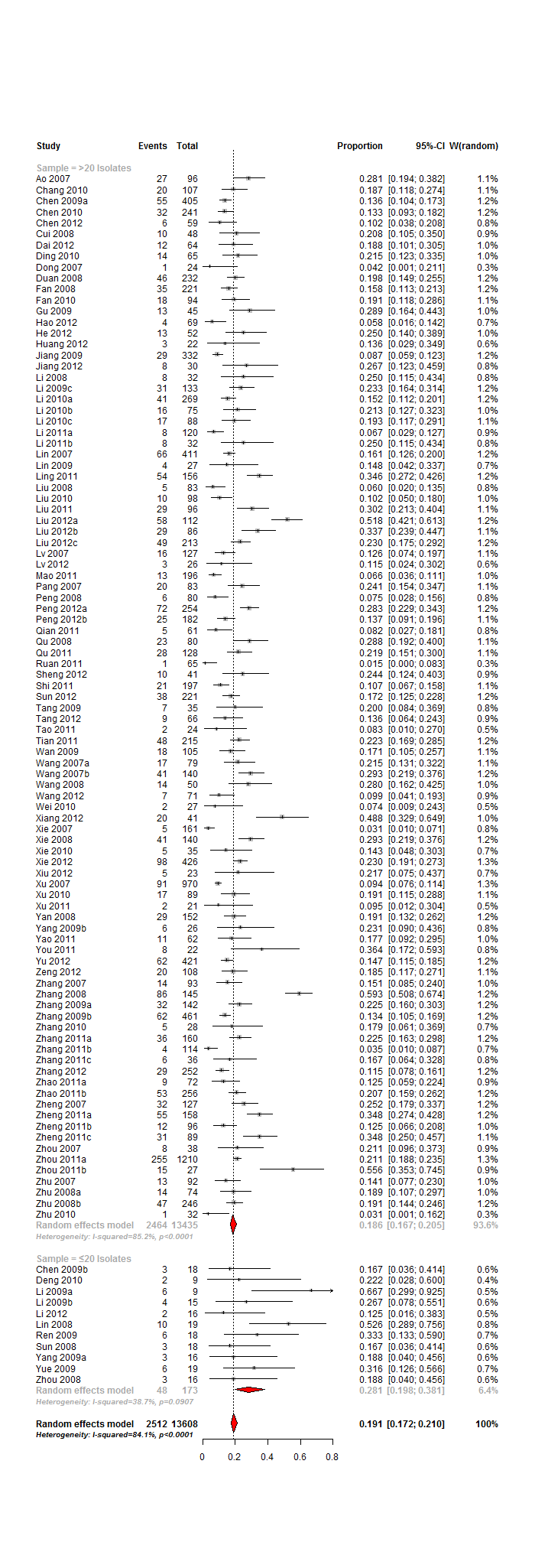


**Figure G. Subgroup analysis for the proportion of *S. aureus* by sample size**


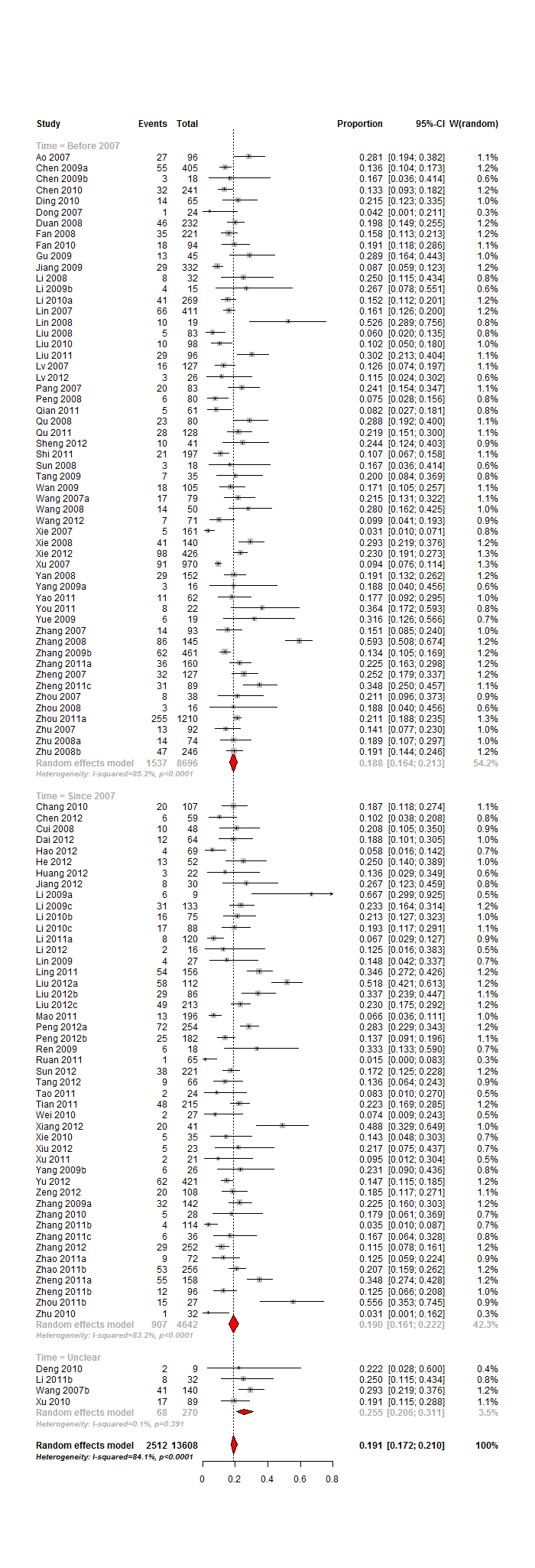


**Figure H. Subgroup analysis for the proportion of *S. aureus* by study onset time**


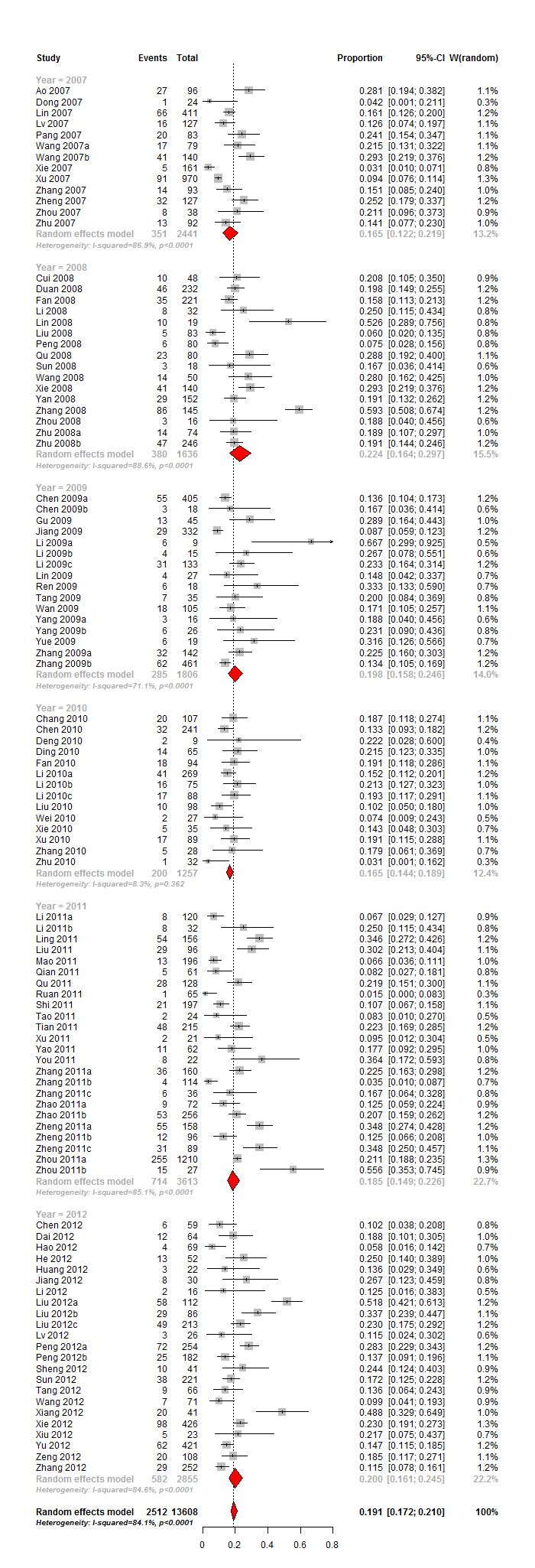


**Figure I. Subgroup analysis for the proportion of *S. aureus* by publication year**


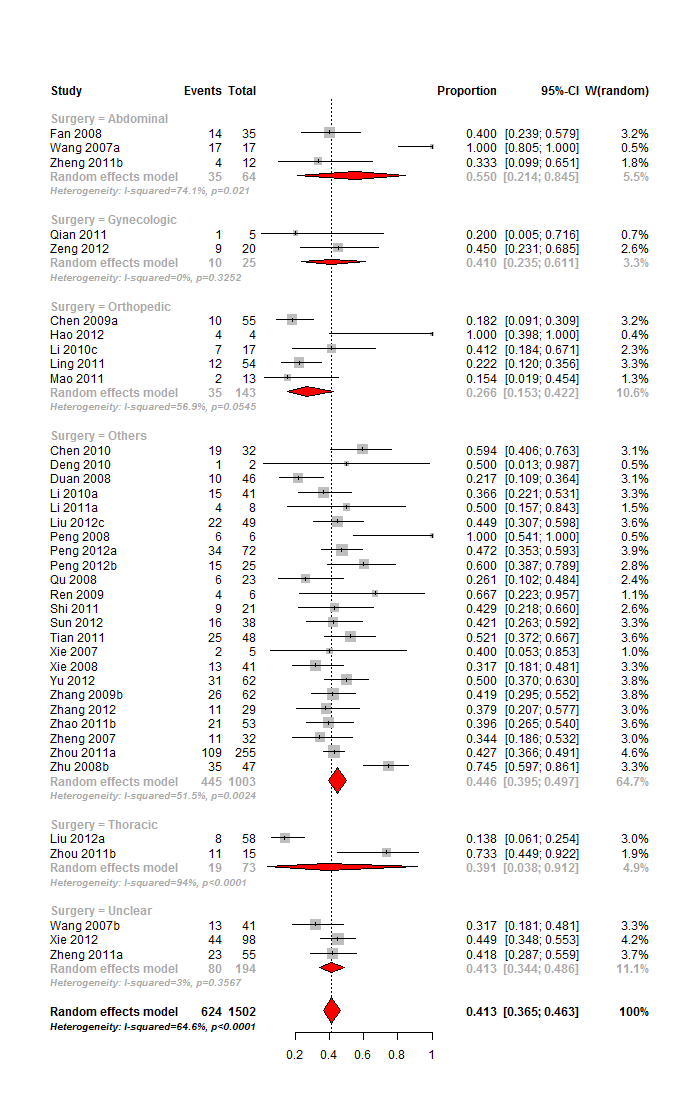


**Figure J. Subgroup analysis for the proportion of MRSA by surgery type**


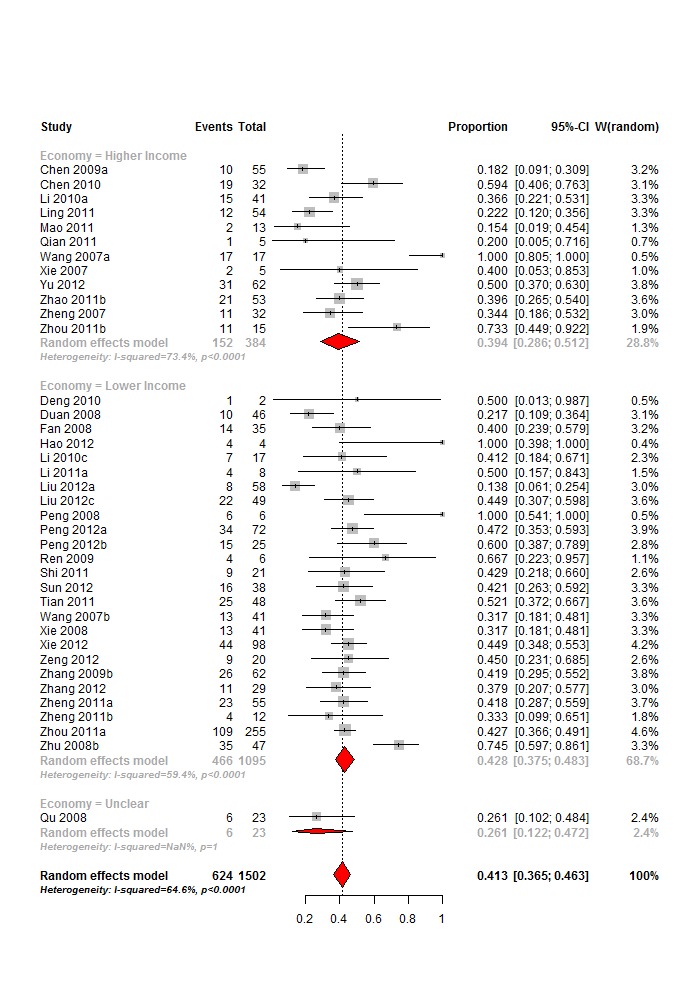


**Figure K. Subgroup analysis for the proportion of MRSA by economic condition**

**
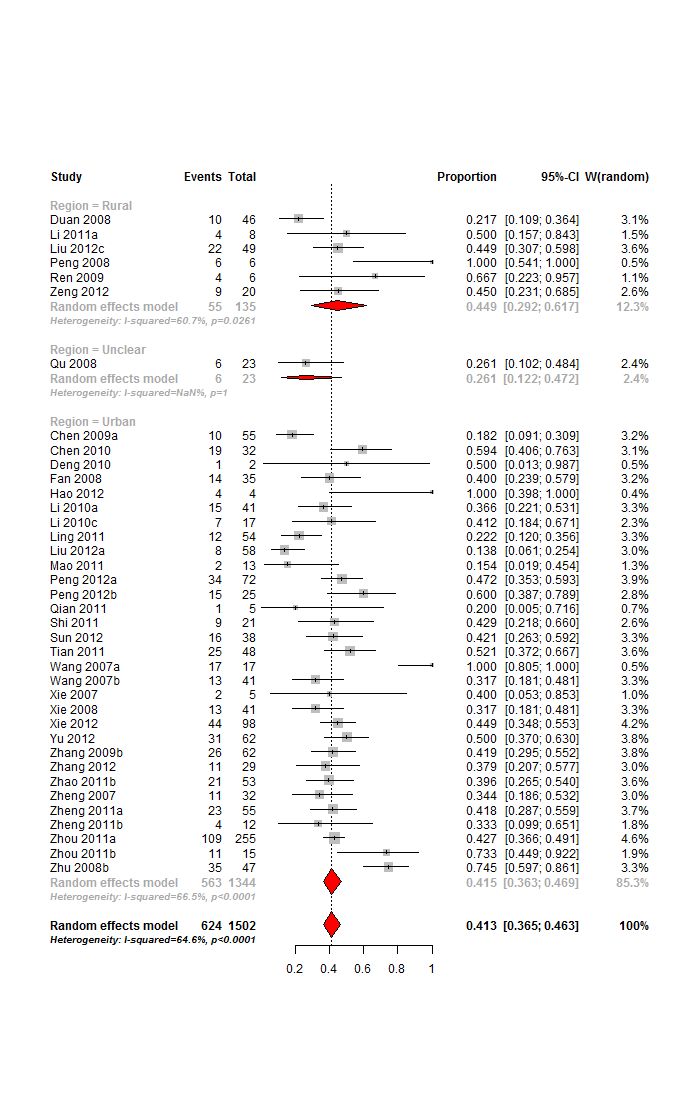
**

**Figure L. Subgroup analysis for the proportion of MRSA by region**

**
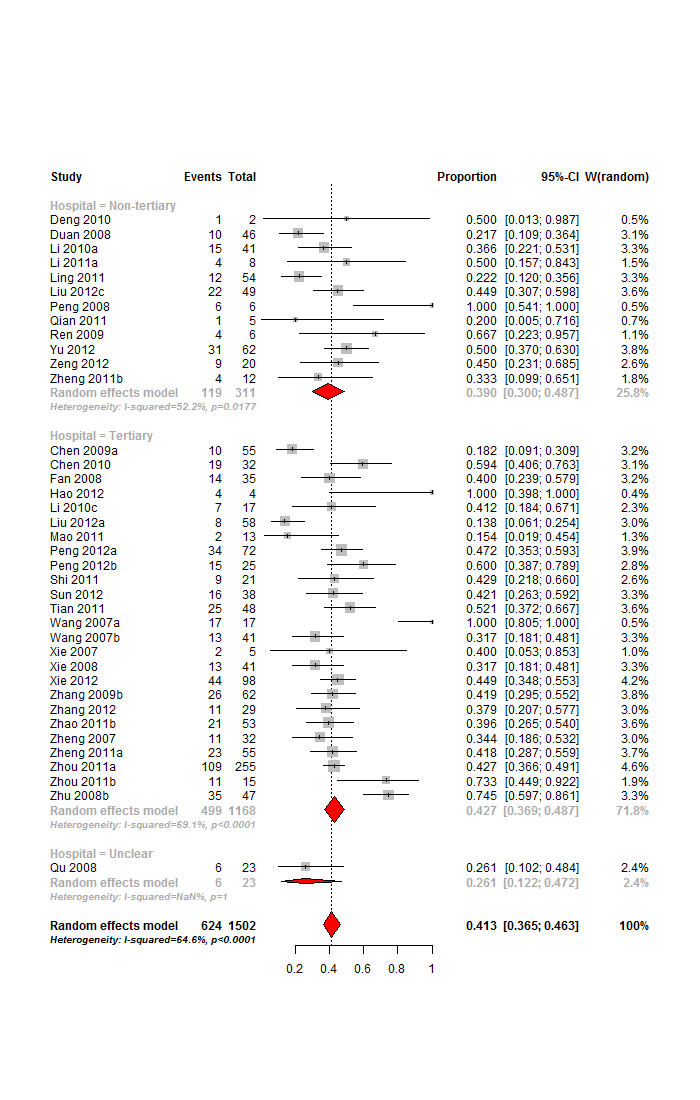
**

**Figure M. Subgroup analysis for the proportion of MRSA by hospital level**

**
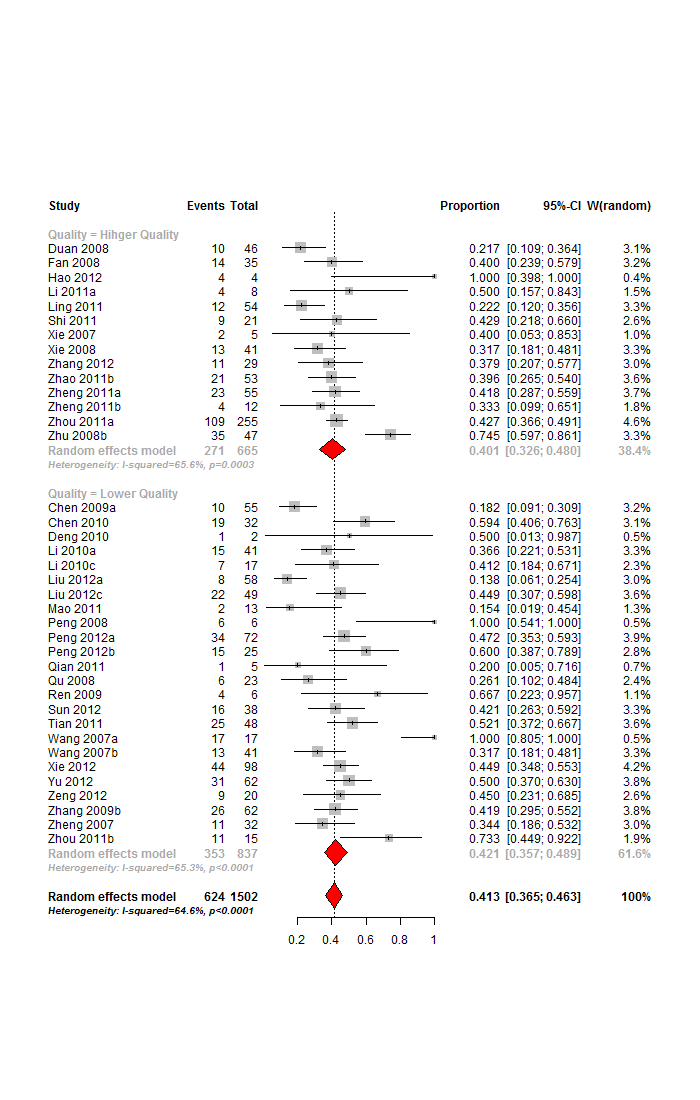
**

**Figure N. Subgroup analysis for the proportion of MRSA by study quality**

**
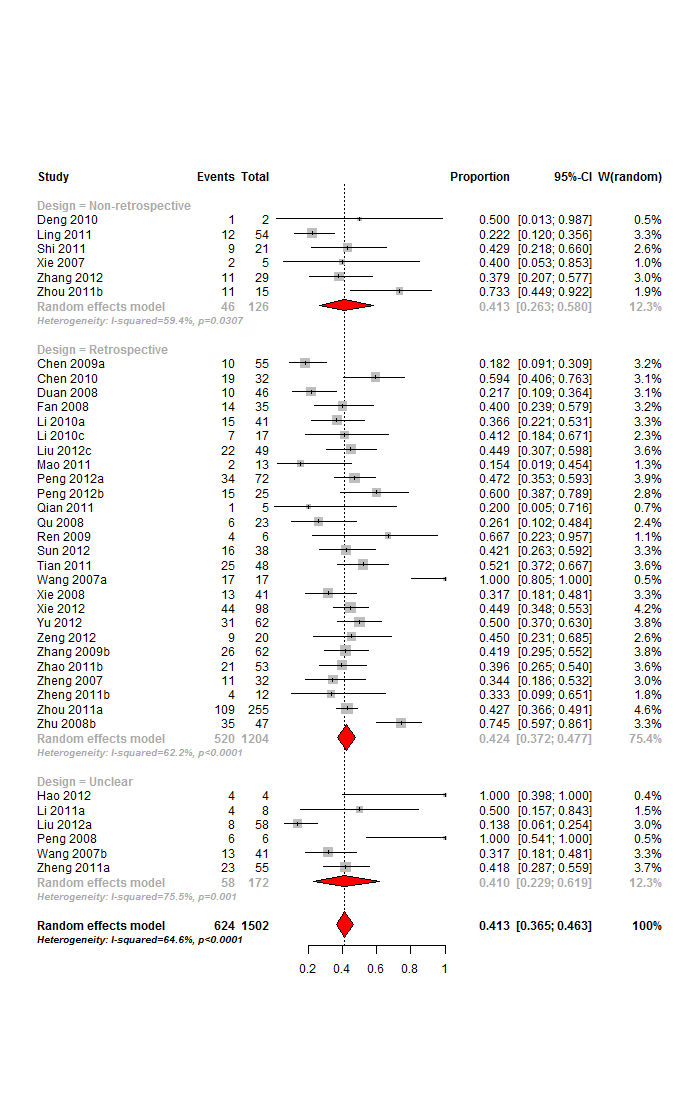
**

**Figure O. Subgroup analysis for the proportion of MRSA by study design**

**
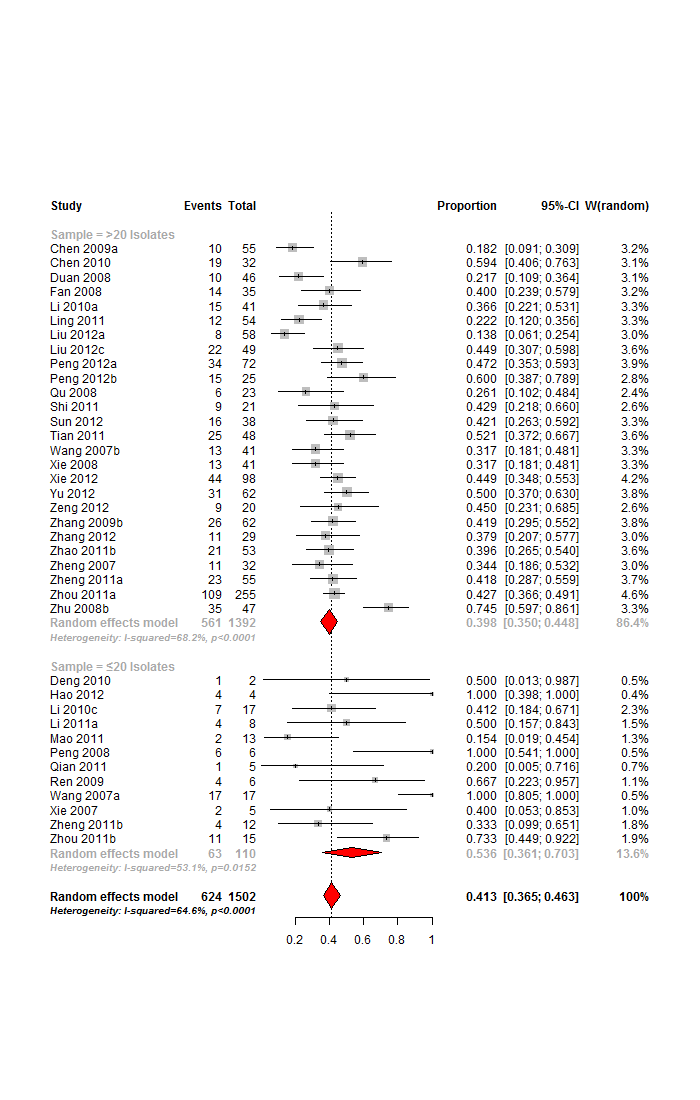
**

**Figure P. Subgroup analysis for the proportion of MRSA by sample size**

**
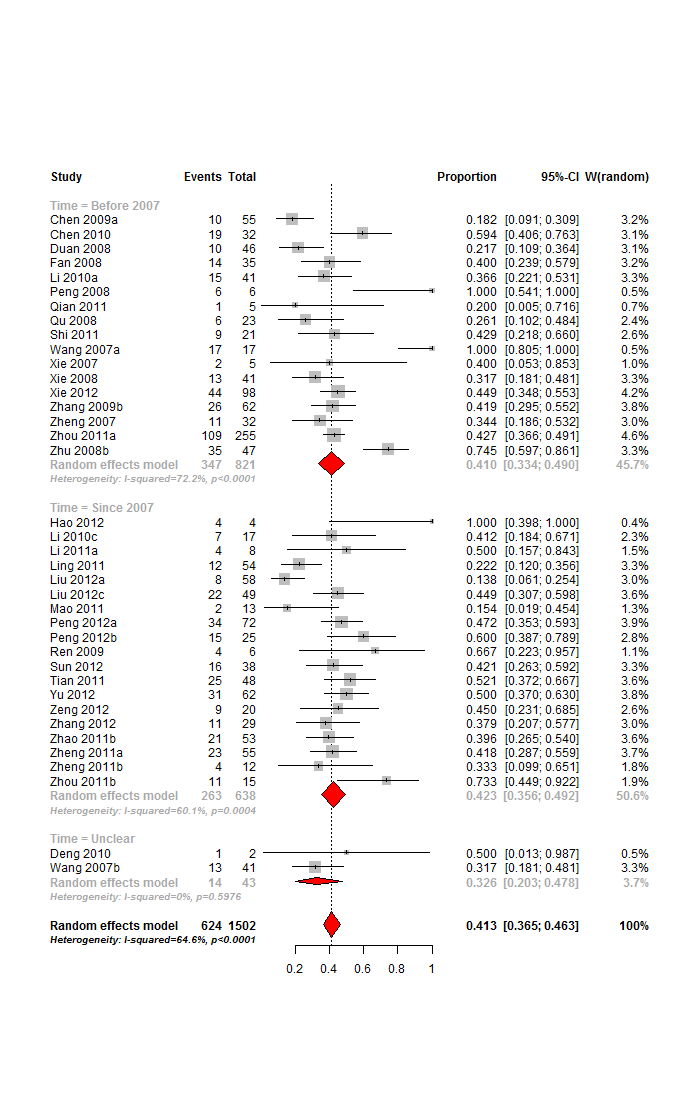
**

**Figure Q. Subgroup analysis for the proportion of MRSA by study onset time**

**
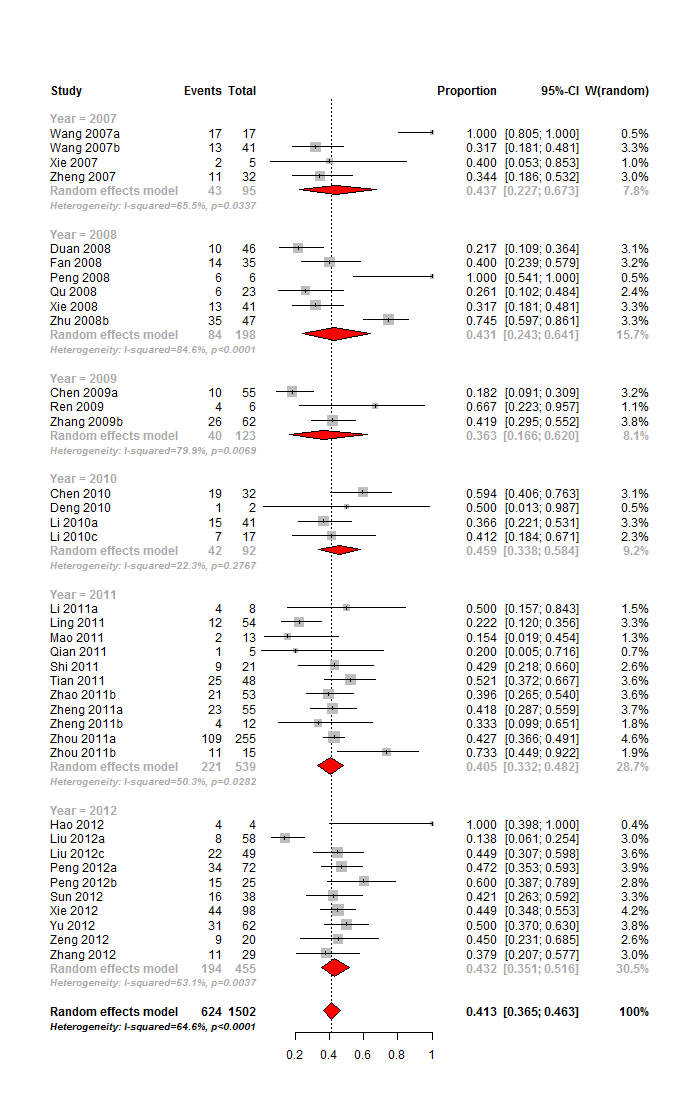
**

**Figure R. Subgroup analysis for the proportion of MRSA by publication year**

**
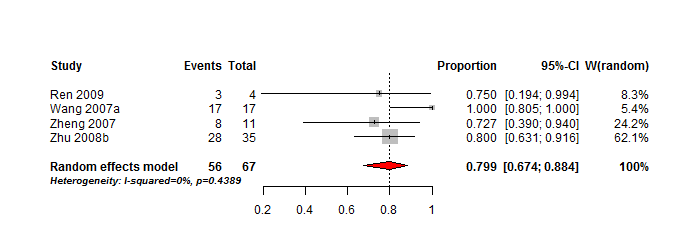
**

**Figure S. Subgroup analysis for the proportion of clindamycin-resistant MRSA**

**
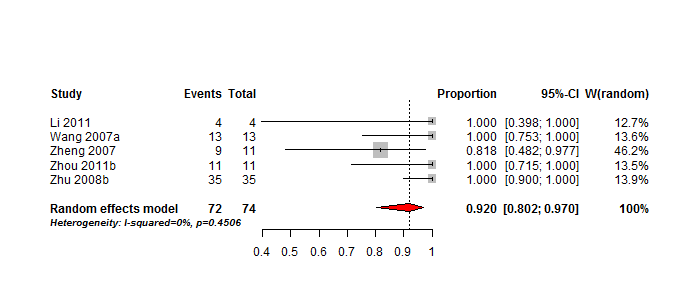
**

**Figure T. Subgroup analysis for the proportion of erythromycin-resistant MRSA**
