# Supplementary material for: Proportions of Staphylococcus aureus and Methicillin-Resistant Staphylococcus aureus in Patients with Surgical Site Infections in Mainland China: A Systematic Review and Meta-Analysis
Source: PLoS One. 2015 Jan 20;10(1):e0116079. doi: 10.1371/journal.pone.0116079 (PMC4300093; doi:10.1371/journal.pone.0116079)
Supplement: S1 Table — (DOCX) [file pone.0116079.s004.docx]

**S1 Table. Search strategies and results**

| Database | Search strategy | Retrieved records |
| --- | --- | --- |
| CBM | ((缺省[智能]:手术 OR 缺省[智能]:术后 OR 缺省[智能]:外科) AND (缺省[智能]:伤口感染 OR缺省[智能]:切口感染 OR 缺省[智能]:创面感染 OR 缺省[智能]:部位感染) AND (缺省[智能]:病原菌 OR 缺省[智能]:病原体 OR 缺省[智能]:耐药 OR 缺省[智能]:药敏 OR 缺省[智能]:菌种 OR缺省[智能]:菌株 OR 缺省[智能]:细菌 OR 缺省[智能]:金黄色葡萄球菌 OR 缺省[智能]:金葡菌 OR 缺省[智能]:甲氧西林)) 限定:2007-2012 | 906 |
| CNKI | (主题="手术"+"术后"+"外科") AND (主题="伤口感染"+"切口感染"+"创面感染"+"部位感染"+"腔隙感染"+"器官感染") AND (主题="病原菌"+"病原体"+"耐药"+"药敏"+"菌株"+"菌种"+"细菌"+"金黄色葡萄球菌"+"金葡菌"+"甲氧新林") AND (年="2007"+"2008"+"2009"+"2010"+"2011"+"2012") | 752 |
| VIP | ((((((文摘=病原菌+菌种)+(文摘=病原体+细菌))+(文摘=耐药+金黄色葡萄球菌))+(文摘=药敏+金葡菌))+(文摘=菌株+甲氧西林)*(医药卫生)*全部期刊*年=2007-2012)*((文摘=伤口感染+切口感染+创面感染+部位感染+器官感染+腔隙感染)*Year=2007-2012))*((文摘=手术+术后+外科)*Year=2007-2012) | 419 |
| Wanfang | (主题:("手术"+"术后"+"外科")) AND (主题:("伤口感染"+"切口感染"+"创面感染"+"部位感染"+"器官感染"+"腔隙感染")) AND (主题:("病原菌"+"病原体"+"耐药"+"药敏"+"菌株"+"菌种"+"细菌"+"金黄色葡萄球菌"+"金葡菌"+"甲氧新林"))*Date:2007-2012 | 737 |
| Pubmed | ((((((((((Staphylococcus aureus[MeSH Terms]) OR ((MRSA[MeSH Terms]) OR MSSA[MeSH Terms])) OR methicillin[MeSH Terms]) OR S. aureus[MeSH Terms]) OR Staphylococcus aureus[Title/Abstract]) OR ((MRSA[Title/Abstract]) OR MSSA[Title/Abstract])) OR methicillin[Title/Abstract]) OR S. aureus[Title/Abstract])) AND ((("cross infection"[MeSH Terms] OR Nosocomial Infection[Text Word] OR hospital infection[text word])) OR ((((Surgery[MeSH Terms]) OR Surgery[Title/Abstract])) AND ((((((wound infection[MeSH Terms]) OR postoperative wound infection[MeSH Terms]) OR infections, surgical wound[MeSH Terms]) OR wound infection[Title/Abstract]) OR postoperative wound infection[Title/Abstract]) OR surgical site infection[Title/Abstract])))) AND ((((((China[MeSH Terms]) OR Chinese[MeSH Terms]) OR mainland China[MeSH Terms]) OR China[Title/Abstract]) OR Chinese[Title/Abstract]) OR mainland [Title/Abstract]) Filters: Publication date from 2007/01/01 to 2012/11/30 | 37 |
| Embase  (OVID) | (('staphylococcus aureus'/exp OR 'staphylococcus aureus' OR 'mrsa'/exp OR 'mrsa' OR 'mssa'/exp OR 'mssa' OR 'methicillin'/exp OR methicillin OR 's. aureus') AND ((('wound infection'/exp OR 'wound infection' OR 'hospital infection'/exp OR 'hospital infection' OR 'nosocomial infection'/exp OR 'nosocomial infection' OR'cross infection'/exp OR 'cross infection')AND ('surgery'/exp OR surgery)) OR (#9'surgical wound infection'/exp OR 'surgical wound infection' OR 'postoperative wound infection'/exp OR 'postoperative wound infection')) AND (mainland OR 'chinese'/exp OR chinese OR 'china'/exp OR 'china')) AND (2007:py OR 2008:py OR 2009:py OR 2010:py OR 2011:py OR 2012:py) | 53 |
