# Supplementary material for: Proportions of Staphylococcus aureus and Methicillin-Resistant Staphylococcus aureus in Patients with Surgical Site Infections in Mainland China: A Systematic Review and Meta-Analysis
Source: PLoS One. 2015 Jan 20;10(1):e0116079. doi: 10.1371/journal.pone.0116079 (PMC4300093; doi:10.1371/journal.pone.0116079)
Supplement: S2 Table — (DOCX) [file pone.0116079.s005.docx]

**Table S2. Quality assessment of included studies**

| **Study ID** | **Specified diagnostic criteria of original diseases** | **Specified diagnostic criteria of SSIs** | **Specified criteria of inclusion** | **Case source** | **Case selection method** | **Specified test for pathogenic bacteria** | **Pathogenic test for all included cases** | **Study type** | **Total scores** |
| --- | --- | --- | --- | --- | --- | --- | --- | --- | --- |
| Ao 2007 | Unclear | Unclear | Unclear | Yes | Consecutive | Yes | Unclear | Retrospective | 3 |
| Chang 2010 | Unclear | Yes | Unclear | Yes | Consecutive | Unclear | Yes | Ambispective | 4 |
| Chen 2009a | Unclear | Unclear | Unclear | Yes | Unclear | Yes | Unclear | Retrospective | 2 |
| Chen 2009b | Unclear | Yes | Unclear | Unclear | Unclear | Unclear | Unclear | Prospective | 2 |
| Chen 2010 | Unclear | Unclear | Unclear | Yes | Unclear | Yes | Yes | Retrospective | 3 |
| Chen 2012 | Unclear | Yes | Unclear | Yes | Consecutive | No | No | Ambispective | 3 |
| Cui 2008 | Unclear | No | Unclear | Yes | Consecutive | Unclear | No | Retrospective | 2 |
| Dai 2012 | Unclear | Yes | Unclear | Yes | Consecutive | No | Unclear | Retrospective | 3 |
| Deng 2010 | Unclear | Yes | Unclear | Unclear | Consecutive | Unclear | No | Cross-sectional | 3 |
| Ding 2010 | Unclear | Unclear | Unclear | Yes | Consecutive | No | Yes | Retrospective | 3 |
| Dong 2007 | Unclear | Unclear | Unclear | Yes | Consecutive | No | No | Monitoring | 3 |
| Duan 2008 | Unclear | Yes | Unclear | Yes | Consecutive | No | Yes | Retrospective | 4 |
| Fan 2008 | Unclear | Yes | Unclear | Yes | Unclear | Yes | Yes | Retrospective | 4 |
| Fan 2010 | Unclear | Unclear | Unclear | Yes | Unclear | No | Yes | Retrospective | 2 |
| Gu 2009 | Unclear | Unclear | Unclear | Unclear | Unclear | No | Unclear | Retrospective | 0 |
| Hao 2012 | Yes | No | Unclear | Yes | Consecutive | No | Yes | Unclear | 4 |
| He 2012 | Unclear | Yes | Unclear | Yes | Consecutive | No | Yes | Monitoring | 5 |
| Huang 2012 | Unclear | Yes | Unclear | Yes | completed data | Yes | Yes | Retrospective | 4 |
| Jiang 2009 | Unclear | Unclear | Unclear | Yes | Unclear | No | Yes | Retrospective | 2 |
| Jiang 2012 | Unclear | Yes | Yes | Yes | Consecutive | No | Yes | Monitoring | 6 |
| Li 2008 | Unclear | No | Unclear | Yes | Unclear | No | Yes | Retrospective | 2 |
| Li 2009a | Unclear | Yes | Unclear | Yes | Consecutive | Unclear | No | Unclear | 3 |
| Li 2009b | Unclear | Unclear | Unclear | Yes | Consecutive | No | Unclear | Retrospective | 2 |
| Li 2009c | Unclear | Yes | Unclear | Yes | Consecutive | No | Yes | Retrospective | 4 |
| Li 2010a | Unclear | Yes | Unclear | Yes | Unclear | Unclear | Yes | Retrospective | 3 |
| Li 2010b | Unclear | Unclear | Unclear | Yes | Consecutive | No | Yes | Retrospective | 3 |
| Li 2010c | Unclear | Unclear | Unclear | Yes | Unclear | Unclear | No | Retrospective | 1 |
| Li 2011a | Unclear | Unclear | Unclear | Yes | Consecutive | Yes | Yes | Unclear | 4 |
| Li 2011b | Unclear | Yes | Unclear | Unclear | Unclear | No | Yes | Unclear | 2 |
| Li 2012 | Unclear | Yes | Unclear | Yes | Consecutive | Unclear | Yes | Retrospective | 4 |
| Lin 2007 | Unclear | Unclear | Unclear | Yes | Consecutive | No | Yes | Unclear | 3 |
| Lin 2008 | Unclear | Yes | Unclear | Yes | Consecutive | Unclear | Yes | Ambispective | 4 |
| Lin 2009 | Unclear | Unclear | Unclear | Yes | Consecutive | Unclear | Yes | Retrospective | 3 |
| Ling 2011 | Unclear | Unclear | Unclear | Unclear | Consecutive | Yes | Yes | Monitoring | 4 |
| Liu 2008 | Unclear | Yes | Unclear | Yes | Consecutive | Yes | Unclear | Ambispective | 4 |
| Liu 2010 | Unclear | Yes | Unclear | Yes | Consecutive | Unclear | Yes | Ambispective | 4 |
| Liu 2011 | Unclear | Yes | Unclear | Yes | Consecutive | Unclear | Unclear | Ambispective | 3 |
| Liu 2012a | Unclear | Unclear | Unclear | Yes | Unclear | No | Yes | Unclear | 2 |
| Liu 2012b | Unclear | Yes | Unclear | Yes | Unclear | Unclear | Yes | Retrospective | 3 |
| Liu 2012c | Unclear | Yes | Unclear | Yes | Consecutive | No | Unclear | Retrospective | 3 |
| Lv 2007 | Unclear | Yes | Unclear | Yes | Consecutive | No | Yes | Retrospective | 4 |
| Lv 2012 | Unclear | Yes | Unclear | Yes | Consecutive | No | Unclear | Retrospective | 3 |
| Mao 2011 | Unclear | Unclear | Unclear | Yes | Consecutive | No | Yes | Retrospective | 3 |
| Pang 2007 | Unclear | Unclear | Unclear | Yes | Consecutive | Yes | Yes | Retrospective | 4 |
| Peng 2008 | Unclear | Unclear | Unclear | Unclear | Consecutive | Unclear | Unclear | Unclear | 1 |
| Peng 2012a | Unclear | Yes | Unclear | Yes | Unclear | No | Yes | Retrospective | 3 |
| Peng 2012b | Unclear | Unclear | Unclear | Yes | Unclear | Yes | Yes | Retrospective | 3 |
| Qian 2011 | Unclear | Unclear | Unclear | Yes | Consecutive | Yes | No | Retrospective | 3 |
| Qu 2008 | Unclear | Unclear | Unclear | Yes | Consecutive | No | Yes | Retrospective | 3 |
| Qu 2011 | Unclear | Yes | Unclear | Yes | Consecutive | Unclear | Yes | Retrospective | 4 |
| Ren 2009 | Unclear | Unclear | Unclear | Yes | Unclear | No | Yes | Retrospective | 2 |
| Ruan 2011 | Unclear | Yes | Yes | Yes | Consecutive | Unclear | No | Retrospective | 4 |
| Sheng 2012 | Unclear | Unclear | Unclear | Yes | Consecutive | No | Unclear | Retrospective | 2 |
| Shi 2011 | Unclear | Yes | Unclear | Yes | Consecutive | Unclear | Yes | Prospective | 5 |
| Sun 2008 | Unclear | Unclear | Unclear | Yes | Unclear | No | Yes | Unclear | 2 |
| Sun 2012 | Unclear | Unclear | Unclear | Yes | Unclear | No | Yes | Retrospective | 2 |
| Tang 2009 | Unclear | Unclear | Unclear | Unclear | Consecutive | Yes | Unclear | Unclear | 2 |
| Tang 2012 | Unclear | Unclear | Unclear | Yes | Consecutive | Unclear | Yes | Retrospective | 3 |
| Tao 2011 | Unclear | Yes | Unclear | Yes | Consecutive | Unclear | Unclear | Ambispective | 3 |
| Tian 2011 | Unclear | Yes | Unclear | Yes | Unclear | No | Yes | Retrospective | 3 |
| Wan 2009 | Unclear | Unclear | Unclear | Yes | Consecutive | No | Yes | Unclear | 3 |
| Wang 2007a | Unclear | Yes | Unclear | Yes | Unclear | Unclear | Unclear | Retrospective | 2 |
| Wang 2007b | Unclear | Unclear | Unclear | Unclear | Unclear | No | Yes | Unclear | 1 |
| Wang 2008 | Unclear | Unclear | Unclear | Yes | Unclear | Unclear | Yes | Unclear | 2 |
| Wang 2012 | Unclear | Yes | Yes | Yes | Unclear | No | Yes | Retrospective | 4 |
| Wei 2010 | Unclear | Yes | Yes | Yes | Consecutive | Yes | Yes | Monitoring | 7 |
| Xiang 2012 | Unclear | No | Unclear | Yes | Consecutive | No | Yes | Retrospective | 3 |
| Xie 2007 | Unclear | Yes | Unclear | Yes | Consecutive | No | No | Prospective | 4 |
| Xie 2008 | Unclear | Unclear | Unclear | Yes | Consecutive | Yes | Yes | Retrospective | 4 |
| Xie 2010 | Unclear | Unclear | Yes | Yes | Consecutive | Unclear | Yes | Cross-sectional | 5 |
| Xie 2012 | Unclear | Unclear | Unclear | Yes | Consecutive | No | Unclear | Retrospective | 2 |
| Xiu 2012 | Unclear | Yes | Unclear | Yes | Unclear | Yes | Yes | Retrospective | 4 |
| Xu 2007 | Unclear | Unclear | Unclear | Yes | Unclear | No | Yes | Retrospective | 2 |
| Xu 2010 | Unclear | Yes | Unclear | Unclear | Unclear | Yes | Yes | Prospective | 4 |
| Xu 2011 | Unclear | Yes | Unclear | Yes | Consecutive | No | Unclear | Retrospective | 3 |
| Yan 2008 | Unclear | Yes | Unclear | Yes | Unclear | Yes | Yes | Unclear | 4 |
| Yang 2009a | Unclear | Yes | Unclear | Yes | Consecutive | No | No | Retrospective | 3 |
| Yang 2009b | Unclear | Yes | Unclear | Yes | Consecutive | Unclear | Yes | Ambispective | 4 |
| Yao 2011 | Unclear | Yes | Unclear | Yes | Unclear | Yes | Yes | Retrospective | 4 |
| You 2011 | Unclear | Yes | Unclear | Yes | Consecutive | Unclear | No | Retrospective | 3 |
| Yu 2012 | Unclear | Unclear | Unclear | Unclear | Consecutive | Yes | Yes | Retrospective | 3 |
| Yue 2009 | Unclear | No | Unclear | Yes | Consecutive | No | Yes | Retrospective | 3 |
| Zeng 2012 | Unclear | Yes | Unclear | Unclear | Unclear | No | Yes | Retrospective | 2 |
| Zhang 2007 | Unclear | Unclear | Unclear | Yes | Unclear | Yes | Yes | Unclear | 3 |
| Zhang 2008 | Unclear | Unclear | Unclear | Yes | Unclear | Unclear | Yes | Unclear | 2 |
| Zhang 2009a | Unclear | Unclear | Unclear | Yes | Consecutive | No | Yes | Retrospective | 3 |
| Zhang 2009b | Unclear | Unclear | Unclear | Unclear | Unclear | Yes | Unclear | Retrospective | 1 |
| Zhang 2010 | Unclear | Yes | Unclear | Yes | Consecutive | No | Yes | Retrospective | 4 |
| Zhang 2011a | Unclear | Unclear | Unclear | Yes | Consecutive | Yes | Yes | Retrospective | 4 |
| Zhang 2011b | Unclear | Yes | Unclear | Yes | Consecutive | Unclear | Unclear | Retrospective | 2 |
| Zhang 2011c | Unclear | Yes | Unclear | Yes | Unclear | No | Yes | Retrospective | 3 |
| Zhang 2012 | Unclear | Yes | Unclear | Yes | Consecutive | Yes | Yes | Ambispective | 5 |
| Zhao 2011a | Unclear | Yes | Unclear | Yes | Consecutive | No | Unclear | Retrospective | 3 |
| Zhao 2011b | Unclear | Yes | Unclear | Yes | Unclear | Yes | Yes | Retrospective | 4 |
| Zheng 2007 | Unclear | Unclear | Unclear | Yes | Consecutive | Unclear | Unclear | Retrospective | 2 |
| Zheng 2011a | Unclear | Yes | Unclear | Yes | Consecutive | No | Yes | Unclear | 4 |
| Zheng 2011b | Unclear | Yes | Unclear | Yes | Consecutive | Yes | Yes | Retrospective | 5 |
| Zheng 2011c | Unclear | Unclear | Yes | Yes | Unclear | No | Yes | Cross-sectional | 4 |
| Zhou 2007 | Unclear | Unclear | Unclear | Yes | Unclear | No | Yes | Retrospective | 2 |
| Zhou 2008 | Unclear | Yes | Unclear | Yes | Unclear | No | Yes | Retrospective | 3 |
| Zhou 2011a | Unclear | Yes | Unclear | Yes | Consecutive | Yes | Yes | Retrospective | 5 |
| Zhou 2011b | Unclear | Unclear | Yes | Unclear | Unclear | No | Yes | Prospective | 3 |
| Zhu 2007 | Unclear | Yes | Unclear | Yes | Consecutive | Unclear | Unclear | Retrospective | 3 |
| Zhu 2008a | Unclear | Yes | Unclear | Yes | Unclear | Yes | Yes | Retrospective | 4 |
| Zhu 2008b | Unclear | Unclear | Unclear | Yes | Consecutive | Yes | Yes | Retrospective | 4 |
| Zhu 2010 | Unclear | Unclear | Unclear | Yes | Consecutive | Yes | Unclear | Unclear | 3 |

Criteria for scoring:

Adapting the scale by NICE, we developed scale for evaluating study quality for each included study with 8 items.

1. Specified diagnostic criteria of original diseases: if original diseases which refer to the causal diseases requiring surgery were specified, we regarded it as low risk of bias and scored 1 point.

2. Specified diagnostic criteria of SSIs: if yes, we regarded it as low risk of bias and scored 1 point.

3. Specified criteria of inclusion: if sufficient information was provided for our understanding of how the patients were included, we regarded it as low risk of bias and scored 1 point.

4. Case source: if time interval and venue of recruiting cases were specified, we regarded it as low risk of bias and scored 1 point.

5. Case selection method: if Consecutive or randomly selective cases were included, we regarded it as low risk of bias and scored 1 point.

6. Specified test for pathogenic bacteria: if valid tests were described in details, we regarded it as low risk of bias and scored 1 point.

7. Pathogenic test for all included cases: if all included cases with SSIs were tested for bacteria, we regarded it as low risk of bias and scored 1 point.

8. Study type: if study design was Prospective, cross-sectional or surveillance, we regarded it as low risk of bias and scored 1 point.

We defined studies with at least 4 points as relatively high-quality studies.
