# Supplementary material for: Proportions of Staphylococcus aureus and Methicillin-Resistant Staphylococcus aureus in Patients with Surgical Site Infections in Mainland China: A Systematic Review and Meta-Analysis
Source: PLoS One. 2015 Jan 20;10(1):e0116079. doi: 10.1371/journal.pone.0116079 (PMC4300093; doi:10.1371/journal.pone.0116079)
Supplement: S3 Table — (DOCX) [file pone.0116079.s006.docx]

**S3 Table. Distribution of MRSA isolates resistant to specific antibiotics**

| **Characteristics** | | **MRSA resistant to vancomycin** | | |  | **MRSA resistant to linezolid** | | |  | **MRSA resistant to clindamycin** | | |  | **MRSA resistant to erythromycin** | | |
| --- | --- | --- | --- | --- | --- | --- | --- | --- | --- | --- | --- | --- | --- | --- | --- | --- |
|  |  | Number of studies | Number of vancomycin -resistant MRSA | Number of MRSA |  | Number of studies | Number of linezolid-resistant MRSA | Number of MRSA |  | Number of studies | Number of clindamycin-  resistant MRSA | Number of MRSA |  | Number of studies | Number of erythromycin -resistant MRSA | Number of MRSA |
| Publication year | 2007 | 2 | 0 | 28 |  | 1 | 1 | 10 |  | 2 | 25 | 28 |  | 2 | 22 | 24 |
|  | 2008 | 6 | 0 | 84 |  | 0 | 0 | 0 |  | 1 | 28 | 35 |  | 1 | 35 | 35 |
|  | 2009 | 3 | 0 | 40 |  | 2 | 0 | 30 |  | 1 | 3 | 4 |  | 0 | 0 | 0 |
|  | 2010 | 1 | 0 | 19 |  | 0 | 0 | 0 |  | 0 | 0 | 0 |  | 0 | 0 | 0 |
|  | 2011 | 8 | 0 | 198 |  | 2 | 0 | 13 |  | 0 | 0 | 0 |  | 2 | 15 | 15 |
|  | 2012 | 8 | 0 | 144 |  | 2 | 0 | 35 |  | 0 | 0 | 0 |  | 0 | 0 | 0 |
| Surgery type | Orthopedic | 2 | 0 | 12 |  | 3 | 0 | 16 |  | 0 | 0 | 0 |  | 0 | 0 | 0 |
|  | Abdominal | 0 | 0 | 0 |  | 1 | 1 | 10 |  | 1 | 17 | 17 |  | 1 | 13 | 13 |
|  | Gynecologic | 0 | 0 | 0 |  | 0 | 0 | 0 |  | 0 | 0 | 0 |  | 0 | 0 | 0 |
|  | Thoracic | 2 | 0 | 19 |  | 1 | 0 | 11 |  | 0 | 0 | 0 |  | 1 | 11 | 11 |
|  | Others* | 17 | 0 | 368 |  | 2 | 0 | 57 |  | 3 | 39 | 50 |  | 3 | 48 | 50 |
|  | Unclear | 2 | 0 | 67 |  | 0 | 0 | 0 |  | 0 | 0 | 0 |  | 0 | 0 | 0 |
| Study design | Retrospective | 20 | 0 | 443 |  | 5 | 1 | 86 |  | 4 | 56 | 67 |  | 3 | 57 | 59 |
|  | Prospective | 2 | 0 | 23 |  | 1 | 0 | 11 |  | 0 | 0 | 0 |  | 1 | 11 | 11 |
|  | Ambispective | 1 | 0 | 11 |  | 0 | 0 | 0 |  | 0 | 0 | 0 |  | 0 | 0 | 0 |
|  | Cross-sectional | 0 | 0 | 0 |  | 0 | 0 | 0 |  | 0 | 0 | 0 |  | 0 | 0 | 0 |
|  | Unclear | 5 | 0 | 45 |  | 1 | 0 | 4 |  | 0 | 0 | 0 |  | 1 | 4 | 4 |
| Region | Urban | 21 | 0 | 461 |  | 7 | 0 | 94 |  | 3 | 53 | 63 |  | 4 | 68 | 70 |
|  | Rural | 6 | 0 | 55 |  | 0 | 0 | 0 |  | 1 | 3 | 4 |  | 1 | 4 | 4 |
|  | Unclear | 1 | 0 | 6 |  | 0 | 0 | 0 |  | 0 | 0 | 0 |  | 0 | 0 | 0 |
| Hospital | Tertiary | 18 | 0 | 418 |  | 6 | 0 | 63 |  | 3 | 53 | 63 |  | 4 | 68 | 70 |
|  | Non-tertiary | 8 | 0 | 98 |  | 1 | 0 | 31 |  | 1 | 3 | 4 |  | 1 | 4 | 4 |
|  | Unclear | 1 | 0 | 6 |  | 0 | 0 | 0 |  | 0 | 0 | 0 |  | 0 | 0 | 0 |
| Economic Condition | Higher | 9 | 0 | 134 |  | 5 | 1 | 64 |  | 2 | 25 | 28 |  | 3 | 33 | 35 |
|  | Lower | 18 | 0 | 382 |  | 2 | 0 | 30 |  | 2 | 31 | 39 |  | 2 | 39 | 39 |
|  | Unclear | 1 | 0 | 6 |  | 0 | 0 | 0 |  | 0 | 0 | 0 |  | 0 | 0 | 0 |
| Study Quality | Higher | 11 | 0 | 256 |  | 1 | 0 | 4 |  | 1 | 28 | 35 |  | 2 | 39 | 39 |
|  | Lower | 18 | 0 | 266 |  | 6 | 0 | 90 |  | 3 | 28 | 32 |  | 3 | 33 | 35 |
| Sample Size** | >20 isolates | 9 | 0 | 336 |  | 2 | 0 | 57 |  | 1 | 28 | 35 |  | 1 | 37 | 39 |
|  | ≤20 isolates | 19 | 0 | 186 |  | 5 | 1 | 37 |  | 3 | 28 | 32 |  | 4 | 35 | 35 |
| Total |  | 28 | 0 | 522 |  | 7 | 1 | 94 |  | 4 | 56 | 67 |  | 5 | 72 | 74 |

*Others refer to: 1) multiple surgeries involved in the study which cannot be classified into a specific type of surgery or 2) a specific type of surgery, rather than orthopedic, abdominal, gynecologic, or thoracic surgeries, which was reported in a small number of studies.

**Sample size refers to isolates of all identified bacteria for the proportion of *S.aureus*, isolates of all identified *S.aureus* for the proportion of MRSA, and isolates of MRSA for the p
